# The CTLA-4 x OX40 bispecific antibody ATOR-1015 induces anti-tumor effects through tumor-directed immune activation

**DOI:** 10.1186/s40425-019-0570-8

**Published:** 2019-04-11

**Authors:** Anne Månsson Kvarnhammar, Niina Veitonmäki, Karin Hägerbrand, Anna Dahlman, Karin Enell Smith, Sara Fritzell, Laura von Schantz, Mia Thagesson, Doreen Werchau, Kristine Smedenfors, Maria Johansson, Anna Rosén, Ida Åberg, Magnus Winnerstam, Eva Nyblom, Karin Barchan, Christina Furebring, Per Norlén, Peter Ellmark

**Affiliations:** Alligator Bioscience AB, Medicon Village, Scheelevägen 2, 223 81 Lund, Sweden

**Keywords:** CTLA-4, OX40, Regulatory T cell, Solid tumors

## Abstract

**Background:**

The CTLA-4 blocking antibody ipilimumab has demonstrated substantial and durable effects in patients with melanoma. While CTLA-4 therapy, both as monotherapy and in combination with PD-1 targeting therapies, has great potential in many indications, the toxicities of the current treatment regimens may limit their use. Thus, there is a medical need for new CTLA-4 targeting therapies with improved benefit-risk profile.

**Methods:**

ATOR-1015 is a human CTLA-4 x OX40 targeting IgG1 bispecific antibody generated by linking an optimized version of the Ig-like V-type domain of human CD86, a natural CTLA-4 ligand, to an agonistic OX40 antibody. In vitro evaluation of T-cell activation and T regulatory cell (Treg) depletion was performed using purified cells from healthy human donors or cell lines. In vivo anti-tumor responses were studied using human OX40 transgenic (knock-in) mice with established syngeneic tumors. Tumors and spleens from treated mice were analyzed for CD8^+^ T cell and Treg frequencies, T-cell activation markers and tumor localization using flow cytometry.

**Results:**

ATOR-1015 induces T-cell activation and Treg depletion in vitro. Treatment with ATOR-1015 reduces tumor growth and improves survival in several syngeneic tumor models, including bladder, colon and pancreas cancer models. It is further demonstrated that ATOR-1015 induces tumor-specific and long-term immunological memory and enhances the response to PD-1 inhibition. Moreover, ATOR-1015 localizes to the tumor area where it reduces the frequency of Tregs and increases the number and activation of CD8^+^ T cells.

**Conclusions:**

By targeting CTLA-4 and OX40 simultaneously, ATOR-1015 is directed to the tumor area where it induces enhanced immune activation, and thus has the potential to be a next generation CTLA-4 targeting therapy with improved clinical efficacy and reduced toxicity. ATOR-1015 is also expected to act synergistically with anti-PD-1/PD-L1 therapy. The pre-clinical data support clinical development of ATOR-1015, and a first-in-human trial has started (NCT03782467).

**Electronic supplementary material:**

The online version of this article (10.1186/s40425-019-0570-8) contains supplementary material, which is available to authorized users.

## Background

The approval of the anti-CTLA-4 antibody ipilimumab (Yervoy®) in 2011 revolutionized the immuno-oncology (IO) field by significantly improving long-term survival in patients with metastatic melanoma. Since then, six additional checkpoint inhibitors targeting programmed cell death protein 1 (PD-1) or programmed death-ligand 1 (PD-L1) have been approved, and a large number of other IO drugs have entered clinical development. In addition to checkpoint inhibitors, these include costimulatory molecules, e.g. OX40, glucocorticoid-induced TNFR-related protein (GITR), 4-1BB, CD27 and CD40 [1–3].

CTLA-4 is constitutively expressed on T regulatory cells (Tregs) and is upregulated on other T cells upon activation [4, 5]. CTLA-4 is highly upregulated in the tumor microenvironment (TME), particularly on Tregs [6, 7]. Several mechanisms of action of CTLA-4 blocking antibodies have been proposed, including activation of effector T cells by blocking the CTLA-4 pathway and depletion of Tregs via antibody-dependent cellular cytotoxicity (ADCC) or antibody-dependent cellular phagocytosis (ADCP) [6, 8–11]. The relative clinical importance of these mechanisms is still debated [8, 12, 13].

Ipilimumab (IgG1 mAb) as monotherapy is approved for the treatment of advanced melanoma, where it has demonstrated substantial and durable effects in about 15–20% of the patients treated [14, 15]. Ipilimumab is now being tested in other cancer types, including non-small cell lung cancer (NSCLC), renal cell carcinoma (RCC), urothelial carcinoma and prostate cancer. Moreover, ipilimumab in combination with nivolumab (anti-PD-1 mAb) is approved in the US for the treatment of unresectable or metastatic melanoma, advanced RCC, and microsatellite instability high or mismatch repair deficient metastatic colorectal cancer (https://www.cancer.gov/about-cancer/treatment/drugs/ipilimumab). However, CTLA-4 blocking antibodies are associated with severe immune-related adverse events due to a systemic activation of T cells, which limits their use [16, 17].

One approach to improve the response rate and reduce immune-related adverse events of anti-CTLA-4 antibodies is to direct the effect to the tumor using bispecific antibodies (bsAbs) [18]. Therefore, we have developed ATOR-1015, a bispecific CTLA-4 and OX40 targeting antibody. Similar to CTLA-4, OX40 is highly up-regulated on activated T cells, particularly Tregs, in the TME [6, 7, 19]. By targeting two receptors that are overexpressed in the tumor, there is a potential to increase localization to the tumor area compared to monospecific antibodies. This in turn may reduce the risk of systemic T-cell activation and improve the efficacy. Moreover, it has been proposed that combining a checkpoint inhibitor with a T cell costimulatory agonistic antibody may convert a cold tumor into a hot tumor by enhancing T cell expansion and effector functions while controlling the suppressive function of Tregs [20, 21]. Several anti-OX40 mAbs are in clinical development either as monotherapy or in combination with a checkpoint inhibitor [22–24], but except for ATOR-1015, there are no other bsAbs targeting a checkpoint inhibitor and a T cell costimulatory receptor in clinical development.

ATOR-1015 was developed by fusing an optimized version of CD86, one of the natural ligands to CTLA-4, to an agonistic OX40 antibody in an IgG1 format. Herein, we describe the development, characterization and pre-clinical proof-of-concept of ATOR-1015.

## Methods

### Antibodies

ATOR-1015 (anti-CTLA-4 x anti-OX40 bsAb), anti-OX40 mAb, anti-CTLA-4 (anti-GFP x anti-CTLA-4 bsAb) and IgG1 isotype control (anti-GFP) were developed using the proprietary ALLIGATOR-GOLD® library and FIND® optimization technology (Additional file 1: Supplementary Methods). For flow cytometry analysis of human cells, the following anti-human antibodies were used: CD3-PECy5 (clone SP34–2), CD4-APC-H7 (clone RPA-T4), CD25-BV421 (clone 2A3), CD127-FITC (clone HIL-7R-M21), CD134 (OX40)-PE (clone L106) and CD152 (CTLA-4)-APC (clone BNI3) (all from BD) and anti-human IgG-PE (Jackson Immuno-Research).

For flow cytometry analysis of murine cells, the following anti-mouse antibodies were used: CD25-PerCPCy5.5 (clone PC61.5), CD45-APCeFluor780 (clone 30-F11), NK1.1-FITC (clone PK136) and Foxp3-APC (clone FJK-16 s) from eBioscience, CD3-PE (clone 145-2C11), CD3-PECy7 (clone 145-2C11), CD4-BV510 (clone RM4–5), CD4- PECy7 (clone RM4–5), CD8-PE (clone 53–6.7), CD8-PerCPCy5.5 (clone 53–6.7), CD11b-FITC (clone M1/70), CD19-FITC (clone 1D3), CD107a-APC (clone 1D4B), MHC-II-FITC (clone 2G9), Granzyme B-PE (clone GB11) and FVS450 viability stain from BD.

### Cell lines

Chinese hamster ovary (CHO) or human embryonic kidney (HEK) cells were transfected to stably express CTLA-4 and/or OX40. The following cell lines were generated and used: CHO-CTLA-4 (0.1 × 10^6^ receptors/cell), HEK-CTLA-4 (0.8 × 10^6^ receptors/cell), CHO-CTLA-4-OX40 (0.7 × 10^6^ CTLA-4 receptors/cell; 4.8 × 10^6^ OX40 receptors/cell) and CHO-OX40 (1.1 × 10^6^ receptors/cell). Moreover, CHO-FcγRI (0.1 × 10^6^ receptors/cell) were used for crosslinking of ATOR-1015. All cell lines were cultured in RPMI-1640 with Glutamax (Gibco) supplemented with 10% fetal bovine serum (FBS, GE Healthcare), 10 mM HEPES (Gibco), zeocin (250 μg/ml) and/or geneticin (600 μg/ml, Gibco) depending on selection pressure.

MB49 bladder cancer cells (EMD Millipore) were cultured in modified DMEM (high glucose, Glutamax and sodium pyruvate) supplemented with 10% FBS. MC38 colon carcinoma cells (Kerafast) were cultured in RPMI-1640 with Glutamax supplemented with 10% FBS, 10 mM HEPES, 1 mM sodium pyruvate and 0.05 mM 2-mercaptoethanol.

### Binding to target-expressing cells

CHO cells stably transfected to express CTLA-4 or OX40 were used to verify binding of ATOR-1015 to its targets and determine binding potency. Briefly, cells (250,000 cells/well) were stained with serially diluted ATOR-1015 or IgG1 control for 1.5 h at 4 °C. Cells were washed in PBS and stained with a secondary PE-labelled anti-human IgG for 30 min at 4 °C. Thereafter, cells were washed and resuspended in BD CellFix followed by analysis on a FACS Verse and calculation of mean fluorescence intensity (MFI).

### CTLA-4 ligand blocking ELISA

Plates were coated with 0.1 mg/ml CD80-Fc or CD86-Fc (R&D Systems) overnight at 4 °C. ATOR-1015 was serially diluted and mixed with a fixed concentration (320 μg/ml) of biotinylated CTLA-4-mFc (Ancell). The mix was then added to the CD80/CD86-coated wells followed by addition of Streptavidin-HRP and SuperSignal® ELISA Chemiluminiscent substrate (Thermo Fisher Scientific). Binding was detected as luminescence, and the percent inhibition was calculated based on the maximal signal in the absence of ATOR-1015.

### CTLA-4 blockade reporter assay

The ability of ATOR-1015 to block CTLA-4 and increase the co-stimulation and activation of T cells was tested in the CTLA-4 blockade reporter assay (Promega). Briefly, serially diluted ATOR-1015 and IgG1 control were immobilized to plates. Thaw-and-use CTLA-4-expressing Jurkat cells genetically engineered with an IL-2-dependent luciferase reporter were thawed, resuspended in RPMI-1640 supplemented with 10% FBS, and added to the plates. Thereafter, thaw-and-use Raji cells expressing CD80/CD86 and an artificial T cell activator targeting CD3 were thawed and added to the plates. After a 6-h incubation period, Bio-Glo luciferase assay reagent was added and luminescence was measured. The fold increase over the background signal was plotted.

### T-cell activation assays

Leukocyte concentrates were obtained from healthy blood donors at Lund University Hospital, Sweden. Peripheral blood mononuclear cells (PBMC) were purified by Ficoll-Paque Plus (GE Healthcare) density centrifugation. CD3^+^ T cells and CD4^+^ T cells were isolated using the Pan T cell Isolation Kit and CD4^+^ T cell Isolation Kit, respectively (Miltenyi Biotec). Cell purity was routinely > 95%. Cells were cultured in RPMI-1640 with Glutamax supplemented with 10% FBS and 10 mM HEPES.

ATOR-1015 was crosslinked by binding to immobilized CTLA-4. 96-well plates were coated with 5 μg/ml CTLA-4-Fc (Orencia®) and 3 μg/ml anti-CD3 (clone OKT3, eBioscience) overnight at 4 °C. Plates were washed and CD3^+^ T cells added (100,000 cells/well) in the absence or presence of ATOR-1015, the mix of anti-CTLA-4 and anti-OX40 antibodies or IgG1 control. After a 72-h incubation period, the levels of IFN-γ in the supernatants were determined using ELISA (R&D Systems).

ATOR-1015 was also crosslinked using HEK cells expressing CTLA-4 or CHO cells expressing FcγRI. Cells were irradiated and allowed to adhere overnight in 96-well plates (30,000 HEK or 100,000 CHO cells/well) at 37 °C. Plates were washed followed by the addition of anti-CD3 coated beads (clone UCHT-1 and OKT3 (BD), respectively) for suboptimal stimulation and CD4^+^ T cells (100,000 cells/well). T cells were cultured for 72 h in the absence or presence of ATOR-1015, the combination of anti-CTLA-4 and anti-OX40 antibodies or IgG1 control. IL-2 release was measured by ELISA (BD).

### Activation of human Tregs

Tregs were isolated from PBMC using the EasySep Human CD4^+^CD127^low^CD25^+^ Regulatory T Cell Isolation Kit (Stemcell Technologies). Purity was assessed by flow cytometry and was consistently > 90%. To induce target expression, Tregs were cultured at 37 °C for 48 h in RPMI-1640 with Glutamax, 10% FBS and 10 mM HEPES in the presence of Human T-Activator CD3/CD28 Dynabeads (Gibco) according to instructions of the manufacturer. The expression of CTLA-4 and OX40 was analyzed by flow cytometry.

### ADCC reporter assay

ADCC reporter assays (FcγRIIIa-V158 and FcγRIIIa-F158) were obtained from Promega and used according to instructions from the manufacturer. Briefly, effector cells stably expressed the Fcγ receptor and a nuclear factor of activated T cells (NFAT) response element driving expression of a firefly luciferase. Effector cells (75,000 cells/well) were cultured together with target-expressing CHO cells or activated Tregs (15,000 cells/well) in the absence or presence of serially diluted ATOR-1015, monotargeting antibodies (anti-CTLA-4 and anti-OX40) alone or in combination and IgG1 control for 6 h. Effector cell engagement induced by the antibodies was quantified by the luciferase produced and measured as luminescence. The fold increase over background signal was plotted.

### ADCC with primary NK cells

NK cells isolated from PBMC using the EasySep Human NK Cell Isolation Kit (Stemcell Technologies) were used as effector cells. Purity was consistently > 90%. Activated Tregs were used as target cells after washing and removal of magnetic anti-CD3/CD28 beads. NK cells and Tregs were co-cultured at an effector:target cell ratio of 15:1 in RPMI 1640 with Glutamax, 10% ultra-low IgG FBS (Gibco), 1 mM sodium pyruvate and 10 mM HEPES in the absence or presence of serially diluted ATOR-1015, the combination of anti-CTLA-4 and anti-OX40 antibodies and IgG1 control. After 4 h, lactate dehydrogenase (LDH) was measured in the supernatants using the Pierce LDH Cytotoxicity Assay Kit (Thermo Fischer Scientific) Target cell lysis was calculated according to instructions of the manufacturer.

### Mouse models

Human OX40 transgenic (hOX40tg) mice were developed by genOway. Briefly, the extracellular part of the murine OX40 (mOX40) gene was replaced with the human extracellular OX40 coding sequence, keeping the mouse transmembrane and intracellular parts intact. This resulted in the expression of the humanized OX40 coding sequence under the control of the endogenous mOX40 promoter along with the disruption of mOX40 expression. The hOX40tg mice are on a C57BL/6 background. Heterozygous hOX40tg mice were generated by breeding male homozygotes together with female BALB/c mice (Janvier). Homozygous and heterozygous hOX40tg mice and C57BL/6 wildtype (wt) mice as controls were characterized for expression of human and murine OX40 on various immune cells from peripheral blood, spleen, lymph nodes and tumors. Functionality of the hOX40 was tested in a T cell assay.

### In vivo experiments in syngeneic tumor models in hOX40tg mice

Eight to twelve weeks old female hOX40tg mice were injected subcutaneously (sc) on the right hind flank on day 0 with MB49 cells (0.25 × 10^6^ cells) or MC38 cells (0.9 × 10^6^ cells). Vehicle (PBS) or antibodies, including ATOR-1015, monotargeting anti-CTLA-4 and anti-OX40 antibodies, IgG1 control, anti-PD-1 antibody (clone RPM1–14, BioXcell) or surrogate anti-CTLA-4 antibodies (clones 9D9 and 9H10, both BioXcell), were administered intraperitoneally (ip) at indicated time-points. For anti-tumor efficacy studies, tumor volume was measured three times weekly with a caliper and calculated as: (width/2 × length/2 × height/2) × 4π/3. Animals were euthanized when the ethical human endpoints were reached, including tumor volume exceeding 2 cm^3^, tumor ulceration or affected health.

### Statistical analyses

Statistical analyses of in vitro data and tumor growth were performed using non-parametric Mann-Whitney, two-tailed test. Survival curves were tested using the Kaplan-Meier method and compared using the Log-Rank test. All statistical analyses were performed using GraphPad Prism (version 7.01). A *p* value of < 0.05 was considered statistically significant.

## Results

### Generation of ATOR-1015, a bispecific antibody targeting CTLA-4 and OX40

**Fig. 1:**
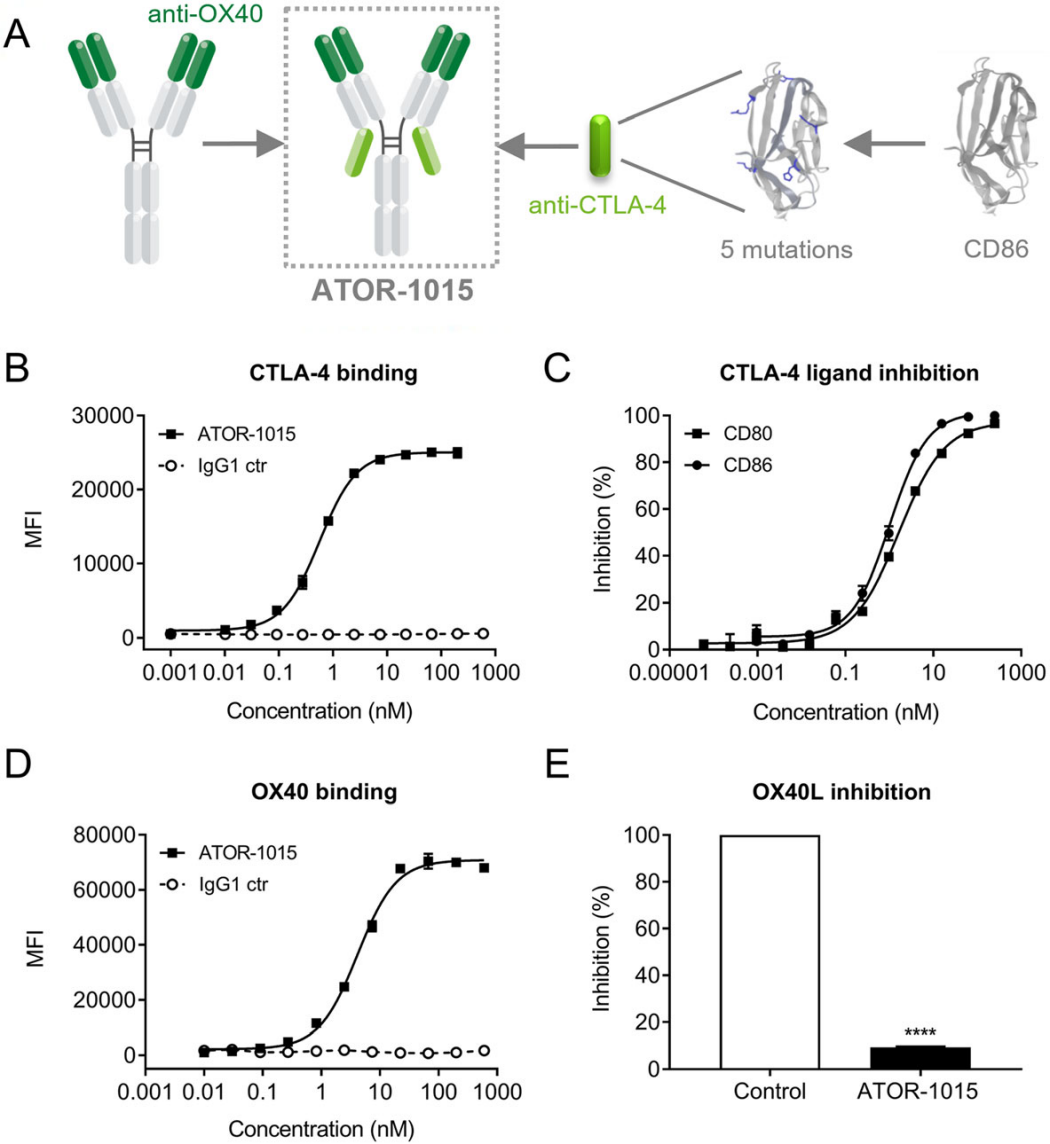
ATOR-1015 binds to CTLA-4 and OX40 and blocks binding to the natural ligands. (**a**) Design of ATOR-1015. The Fab domains bind to OX40. The CTLA-4 binding domains, which are fused to the light chain via a S (GGGGS)_2_ linker, consists of 111 amino acids from CD86 with 5 mutations for enhanced CTLA-4 affinity. (**b**) Binding of ATOR-1015 to CTLA-4-expressing CHO cells. Cells were stained with serially diluted ATOR-1015 or IgG1 control, followed by a PE-conjugated anti-human IgG. Mean fluorescence intensity (MFI) was determined by flow cytometry (*n* = 3). (**c**) ATOR-1015 completely blocks CTLA-4 from interacting with CD80 and CD86 in a competitive ELISA. Plates were coated with CD80-Fc or CD86-Fc. Serially diluted ATOR-1015 was mixed with a fixed concentration of biotinylated CTLA-4-mFc and added to the plates. Streptavidin-HRP and substrate was added, and luminescence was measured. Percent inhibition calculated based on the maximal signal in the absence of ATOR-1015 is shown (*n* = 2). (**d**) Binding of ATOR-1015 to OX40 expressing CHO cells. Cells were stained as described in (B) (n = 2). (**e**) OX40 expressing CHO cells were pre-incubated with or without ATOR-1015 followed by the addition of OX40L and a fluorescently labelled detection antibody. Percent OX40L inhibition was assessed by flow cytometry (n = 3). Statistical analysis was performed using the Mann Whitney, two-tailed test (****, *p* < 0.0001). All data presented as mean ± SEM. *n* equals the number of independent experiments

ATOR-1015 is a human IgG1 bsAb targeting CTLA-4 and OX40. The OX40 binding Fab domains were isolated from the ALLIGATOR-GOLD® human scFv library using phage display technology. The CTLA-4 binding part was generated by improving the stability and affinity of the Ig-like V-type domain of human CD86, one of the natural ligands for CTLA-4, using FIND® and phage display. It consists of a 111 amino acid sequence from CD86 (position 24–124) with 5 mutations that resulted in a ~ 100-fold increased binding to CTLA-4 compared to wildtype CD86 (Additional file 2: Figure S1A), as well as improved developability. The CTLA-4 binding domain was fused to the C-terminal end of the ĸ light chain of the OX40 antibody with a S (GGGGS)_2_ linker (Fig. 1a).

### ATOR-1015 binds to CTLA-4 with high affinity and blocks the interaction with CD80 and CD86

The affinity to CTLA-4 measured using Biacore was determined to 3.0 nM (Additional file 1: Supplementary Methods and Additional file 3: Table S1). Binding to CTLA-4 was tested by flow cytometry using CHO cells stably transfected to express CTLA-4, resulting in an EC_50_ value of 0.7 nM (Fig. 1b). Further, the ability of ATOR-1015 to block the interaction of recombinant CTLA-4 with CD80 and CD86 was tested using ELISA. ATOR-1015 was found to completely inhibit CTLA-4 from interacting with CD80 and CD86 (Fig. 1c). ATOR-1015 also completely blocked CD80 and CD86 from binding to CTLA-4 expressed by CHO cells (Additional file 2: Figure S1B). Moreover, ATOR-1015 did not bind to CD28 as measured by ELISA or flow cytometry (Additional file 2: Figure S1C, D).

### ATOR-1015 binds to domain 2 on OX40 and inhibits the interaction with OX40L

The affinity to OX40 measured using Biacore was determined to 1.6 nM (Additional file 1: Supplementary Methods and Additional file 3: Table S1). Binding to OX40 was further tested using CHO cells stably transfected to express OX40 stained with ATOR-1015, resulting in an EC_50_ value of 2.6 nM (Fig. 1d). A domain mapping was performed using mouse/human chimeric OX40 constructs since ATOR-1015 does not recognize mOX40 (Additional file 1: Supplementary Methods). Loss of binding was observed against chimeras where domain 2 was replaced with its murine counterpart, indicating that ATOR-1015 interacts with domain 2 on OX40. In addition, ATOR-1015 was shown to block OX40L, which also binds to domain 2 on OX40, from interacting with OX40 (Fig. 1e).

### ATOR-1015 can crosslink cells expressing CTLA-4 with cells expressing OX40

**Fig. 2:**
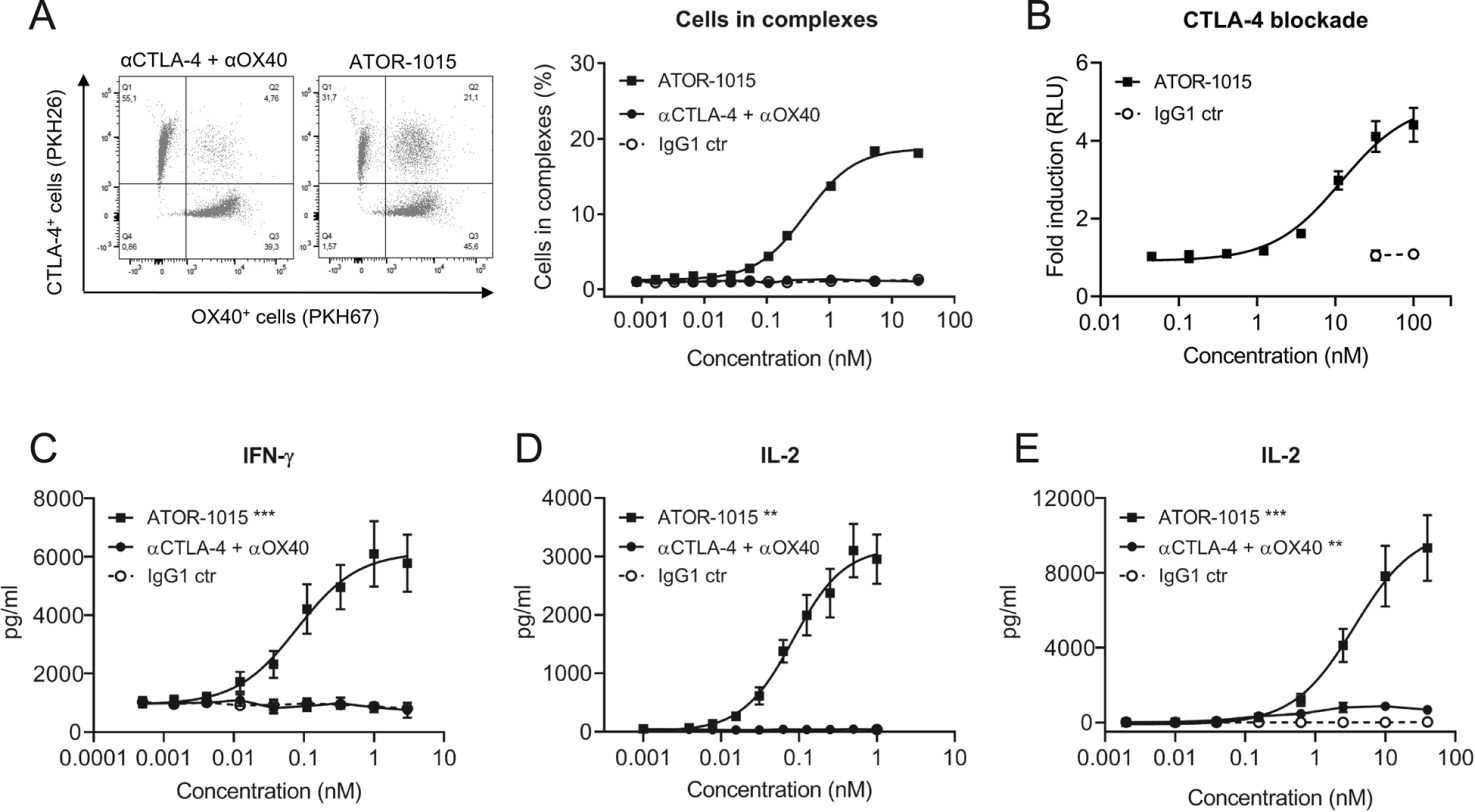
ATOR-1015 generates clustering of cells and induces T-cell activation. (**a**) ATOR-1015, the combination of monotargeting anti-CTLA-4 and anti-OX40 antibodies or IgG1 control was added to a 1:1 mix of PKH26-labelled CTLA-4-expressing HEK cells and PKH67-labelled OX40-expressing CHO cells. The formation of cell complexes was determined by flow cytometry. Data show one representative experiment out of three. (**b**) ATOR-1015 blocks CTLA-4 and promotes T-cell activation in a CTLA-4 blockade reporter assay. Jurkat T cells engineered to express CTLA-4 with an IL-2 dependent luciferase reporter were co-cultured with Raji cells expressing CD80/CD86 and an artificial T cell receptor activator. Serially diluted ATOR-1015 or IgG1 control was added. Upon addition of ATOR-1015, binding of CD80/CD86 to CTLA-4 was prevented, and co-stimulation of T cells via CD28 increased, leading to a luminescent signal. Data presented as fold induction over media control based on relative light units (RLU) (n = 2 independent experiments). (**c**) OX40-mediated T-cell activation upon ATOR-1015 crosslinking to immobilized CTLA-4. Plates were coated with CTLA-4Fc (5 μg/ml) and suboptimal levels of anti-CD3 (OKT3, 3 μg/ml). CD3^+^ T cells (100,000 cells/well) were added along with ATOR-1015, the combination of monotargeting antibodies and IgG1 control. After 72 h of incubation, levels of IFN-γ were measured in the supernatants by ELISA (*n* = 8 donors). (**d**) OX40-mediated T-cell activation upon ATOR-1015 crosslinking to CTLA-4 on cells. HEK cells expressing CTLA-4 (30,000 cells/well) were irradiated and allowed to adhere overnight. CD4^+^ T cells (100,000 cells/well) were added along with anti-CD3 (UCHT1) beads, ATOR-1015, the combination of monotargeting antibodies or IgG1 control. After 72 h of incubation, levels of IL-2 were measured in the supernatants by ELISA (*n* = 6 donors). (**e**) FcγRI-expressing CHO cells (100,000 cells/well) were irradiated and allowed to adhere overnight. CD4^+^ T cells (100,000 cells/well) were added along with anti-CD3 (OKT3) beads, ATOR-1015, the combination of monotargeting antibodies or IgG1 control. After 72 h of incubation, levels of IL-2 were measured in the supernatants by ELISA (n = 8 donors). All data presented as mean ± SEM. Statistical analysis (in C-E) was performed using the Mann Whitney, two-tailed test (**, *p* < 0.01; ***, *p* < 0.001)

To assess whether ATOR-1015 can bind CTLA-4 and OX40 on two different cells simultaneously (i.e. induce formation of cell-cell complexes), CTLA-4-expressing HEK cells dyed with PKH26 and OX40-expressing CHO cells labelled with PKH67 were mixed 1:1 with ATOR-1015, a combination of monotargeting antibodies (anti-CTLA-4 and anti-OX40) or an IgG1 control. In samples incubated with ATOR-1015, there was a dose-dependent increase in the formation of cell complexes, but not in samples incubated with the combination of monotargeting antibodies or the control (Fig. 2a).

### ATOR-1015 induces T-cell activation

The ability of ATOR-1015 to activate T cells by blocking CTLA-4 was tested in a CTLA-4 blockade reporter assay. Jurkat cells engineered to express CTLA-4 and a luciferase reporter driven by an IL-2 promoter responding to TCR/CD28 activation were co-cultured with Raji cells expressing CD80/CD86 and an artificial T cell activator designed to activate the TCR. In the absence of ATOR-1015, CD80/CD86 binds to CTLA-4 with higher affinity than to CD28, thus blocking the signal. By contrast, the addition of increasing concentrations of ATOR-1015 led to a dose-dependent increase in IL-2-driven reporter gene activation, mediated by CD80 and CD86 binding to and activating CD28 (Fig. 2b).

The functional effect of ATOR-1015 on primary human T cell responses was evaluated. ATOR-1015 was designed as a crosslinking-dependent OX40 antibody, requiring crosslinking via either CTLA-4 or Fcγ receptors for induction of T-cell activation. This crosslinking dependency was evaluated in two different assays. Firstly, ATOR-1015 induced a dose-dependent OX40-mediated T-cell activation when crosslinked by CTLA-4, either immobilized (Fig. 2c), or expressed on cells (Fig. 2d). No activation was seen with the combination of monotargeting antibodies or isotype control. Secondly, T-cell activation of ATOR-1015 upon FcγRI crosslinking was demonstrated. ATOR-1015 induced a strong IL-2 release, which was superior to the effect mediated by the combination of monotargeting antibodies (Fig. 2e).

### ATOR-1015 induces depletion of Tregs

**Fig. 3:**
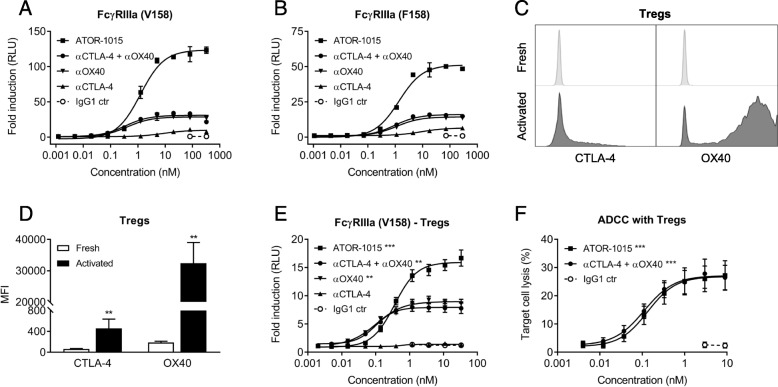
ATOR-1015 induces Fcγ receptor signaling and depletion of target-expressing cells. (**a**, **b**) CHO cells expressing CTLA-4 and OX40 were cultured together with FcγRIIIa (F158 or V158 variant) reporter cells with an NFAT response element driving expression of a firefly luciferase. ATOR-1015, monotargeting anti-CTLA-4 and anti-OX40 antibodies alone or in combination and IgG1 control were added and Fcγ receptor activation was quantified through the luciferase produced and measured as luminescence. Data presented as fold induction based on relative light units (RLU) over media control (n = 2 independent experiments). (**c**) The expression of CTLA-4 and OX40 on the cell surface of freshly isolated and activated (stimulated for 48 h with anti-CD3/CD28 beads) Tregs determined by flow cytometry. Histogram plots from one representative donor are shown. (**d**) The expression of CTLA-4 and OX40 on the cell surface of freshly isolated and activated Tregs. Mean fluorescence intensity (MFI) was measured by flow cytometry (*n* = 5 donors). (**e**) FcγRIIIa reporter cells (V158 variant) were cultured together with activated Tregs. FcγRIIIa activation was detected as described above (n = 5 donors). (**f**) Activated Tregs as target cells were cultured together with allogeneic NK cells as effector cells (effector:target cell ratio 15:1) in the absence or presence of antibodies. After 4 h, target cell lysis was measured based on lactate dehydrogenase (LDH) release (*n* = 7 donors). All data presented as mean ± SEM. Statistical analysis (in D-F) was performed using the Mann Whitney, two-tailed test (**, *p* < 0.01; ***, *p* < 0.001)

The ability of ATOR-1015 to induce ADCC was measured indirectly using reporter assays with FcγRIIIa, both the high (V158) and low affinity (F158) variant. CHO cells transfected to express CTLA-4 and OX40 were used as target cells. In these reporter assays, ATOR-1015 induced strong Fcγ receptor engagement, many-fold higher than the monotargeting antibodies alone or in combination (Fig. 3a, b).

Treg depletion was assessed using primary human Tregs as target cells. Peripheral Tregs express very low levels of CTLA-4 and OX40 [6, 7, 25, 26]. Activation by anti-CD3/CD28 beads led to an up-regulation of both CTLA-4 and OX40, although the increase was more pronounced for OX40 (Fig. 3c, d). The superior FcγRIIIa engagement of ATOR-1015 compared to the monotargeting antibodies alone or in combination was confirmed. A low level of FcγRIIIa engagement was seen with the anti-CTLA-4 antibody, likely due to the relatively low expression of CTLA-4 compared to OX40 (Fig. 3e). Moreover, activated Tregs were used in an ADCC assay with primary NK cells as effector cells. A dose-dependent lysis of Tregs was seen both with ATOR-1015 and the combination of anti-CTLA-4 and anti-OX40 antibodies (Fig. 3f).

### hOX40tg mice express functional hOX40

The CTLA-4 binding domain of ATOR-1015 is fully cross-reactive to murine CTLA-4 (mCTLA-4). However, the OX40 binding Fab does not cross-react with mOX40. Therefore, a hOX40tg mouse was generated to enable in vivo studies of both targets. The hOX40tg mouse model was validated in terms of hOX40 expression and functionality. Briefly, the expression pattern of hOX40 on resting immune cells from blood, spleen and lymph nodes from hOX40tg mice was found to be similar to the expression of mOX40 in wt mice (Additional file 4: Figure S2A). The expression of hOX40 and mOX40 was also tested on splenic CD4^+^ T cells from homozygotes, heterozygotes and wt mice upon stimulation for 48 h with anti-CD3/CD28. The frequency of hOX40^+^ T cells was higher in the homozygous than in the heterozygous mice (50 vs 30–35%). In contrast, almost all CD4^+^ T cells from wt and heterozygous hOX40tg mice expressed mOX40 (Additional file 4: Figure S2B). Next, the expression of hOX40 and mCTLA-4 was assessed on Tregs (CD4^+^ CD25^+^ Foxp3^+^) and conventional T cells (CD4^+^ CD25^−^ Foxp3^−^) from tumors of mice with MC38 cancer. As expected, the levels were higher on Tregs than conventional CD4^+^ T cells (Additional file 4: Figure S2C). Lastly, functionality of the hOX40 receptor was confirmed in a CD4^+^ T cell assay with immobilized CTLA-4 and suboptimal anti-CD3. ATOR-1015, a surrogate bsAb (targeting murine OX40 and CTLA-4) and IgG1 control was added, and levels of IL-2 were measured after 72 h. As expected, only ATOR-1015 was able to induce activation of T cells from the homozygous hOX40tg mice. In heterozygous mice, IL-2 release was seen with both ATOR-1015 and the surrogate bsAb (Additional file 4: Figure S2D).

### ATOR-1015 induces anti-tumor responses and long-term immunological memory

**Fig. 4:**
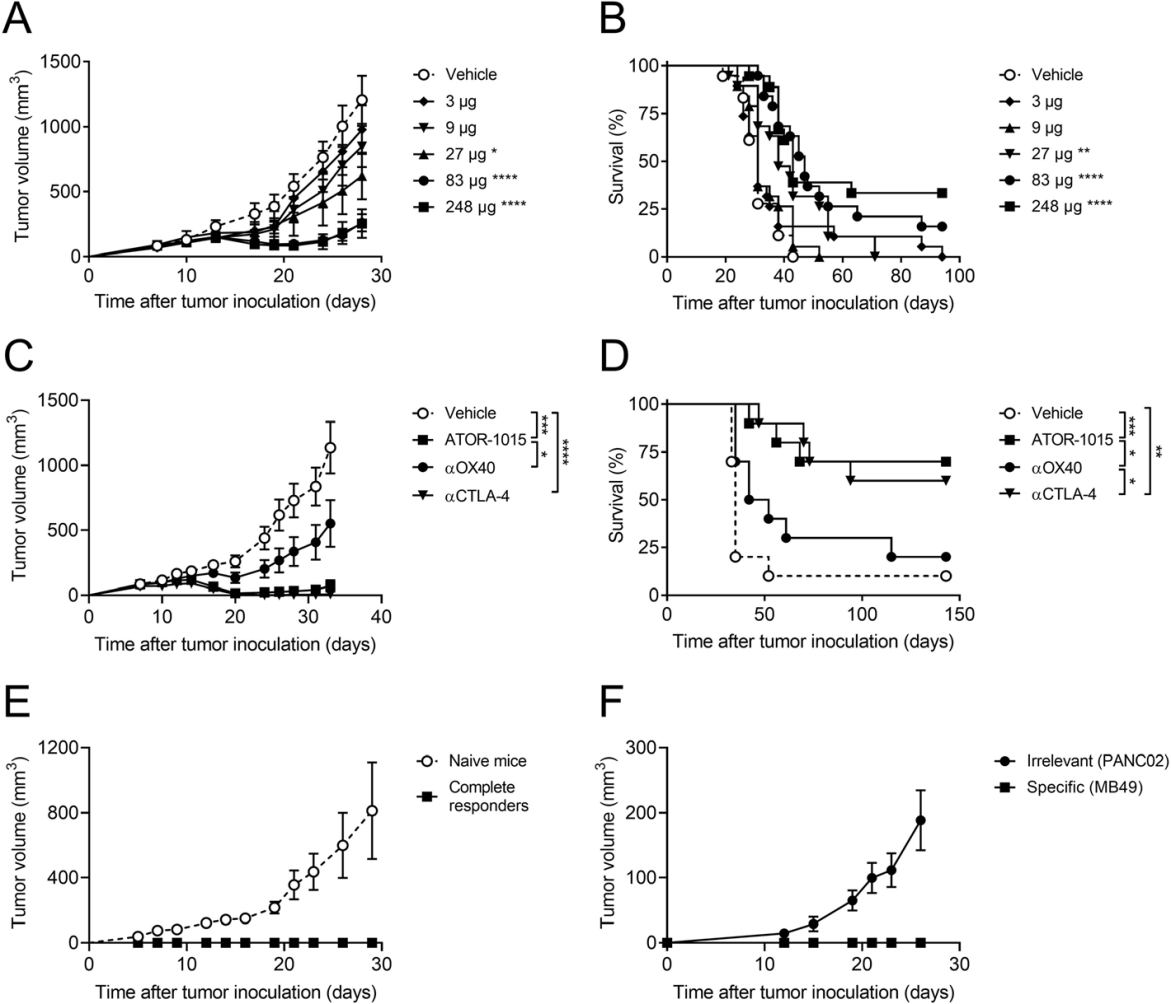
ATOR-1015 induces anti-tumor responses and immunological memory. Female hOX40tg mice were inoculated with MB49 cells day 0 and treated with indicated doses of ATOR-1015, monotargeting anti-CTLA-4 and anti-OX40 antibodies or vehicle day 7, 10 and 13 followed by monitoring of tumor volume and survival. (**a**, **b**) Tumor volume and survival of homozygous mice given vehicle or different doses of ATOR-1015 (*n* = 18–19). (**c**, **d**) Tumor growth and survival in heterozygous mice treated with ATOR-1015, anti-CTLA-4, anti-OX40 antibodies (248 μg for bsAbs or 200 μg for mAbs) or vehicle (*n* = 10). (**e**) Complete responders after treatment with ATOR-1015 as described above were re-exposed with MB49 tumor cells to demonstrate immunological memory. Naïve mice were included as tumor growth controls (n = 5). (**f**) Complete responders after treatment with ATOR-1015 as described above were re-exposed in a twin tumor model with the specific tumor (MB49) in one flank and an irrelevant tumor (PANC02) in the other to demonstrated tumor-specific memory (n = 6). All data is presented as mean ± SEM. Tumor volume was analyzed using Mann-Whitney, two-tailed test and survival using Kaplan-Meier, Log-Rank (*, *p* < 0.05; **, p < 0.01; ***, p < 0.001; ****, *p* < 0.0001). *n* equals the number of mice

The anti-tumor effects of ATOR-1015 were assessed in several syngeneic tumor models in hOX40tg mice. The dose-response relationship was first assessed in the MB49 bladder cancer model. Both homozygous and heterozygous mice were tested. Firstly, MB49 tumor cells were injected sc into the right hind flank of female homozygous mice on day 0. ATOR-1015 or vehicle was administered ip as a flat dose on days 7, 10 and 13 followed by monitoring of tumor growth and survival. Significant anti-tumor responses were seen at doses from 27 μg. Highest effect was seen with the highest dose of 248 μg (Fig. 4a, b).

Next, the effect of ATOR-1015 (248 μg) was compared against the monotargeting anti-CTLA-4 and anti-OX40 antibodies at equimolar doses (corresponding to 200 μg for mAbs) in heterozygous mice. ATOR-1015 and the anti-CTLA-4 antibody reduced tumor growth, prolonged survival and cured tumor-bearing mice (complete responders: 1/10 for vehicle; 2/10 for anti-OX40; 6/10 for anti-CTLA-4; 7/10 for ATOR-1015). Moreover, the anti-tumor effect of ATOR-1015 was superior to the anti-OX40 antibody (Fig. 4c, d).

For assessment of immunological memory, MB49 cells were inoculated sc in the heterozygous mice that previously had been cured from MB49 bladder cancer following treatment with ATOR-1015. Naïve mice were included as controls for tumor growth. It was demonstrated that ATOR-1015 induced immunological memory to MB49, resulting in immunity to tumor re-exposure for at least 5 months (Fig. 4e). Further, the immunity induced by ATOR-1015 was shown to be tumor-specific. Complete responders re-exposed to MB49 and an unspecific tumor not previously encountered (PANC02), demonstrated growth only of the unspecific tumor (Fig. 4f).

### ATOR-1015 is directed to the tumor and increases intratumoral CD8^+^ T cell/Treg ratios

**Fig. 5:**
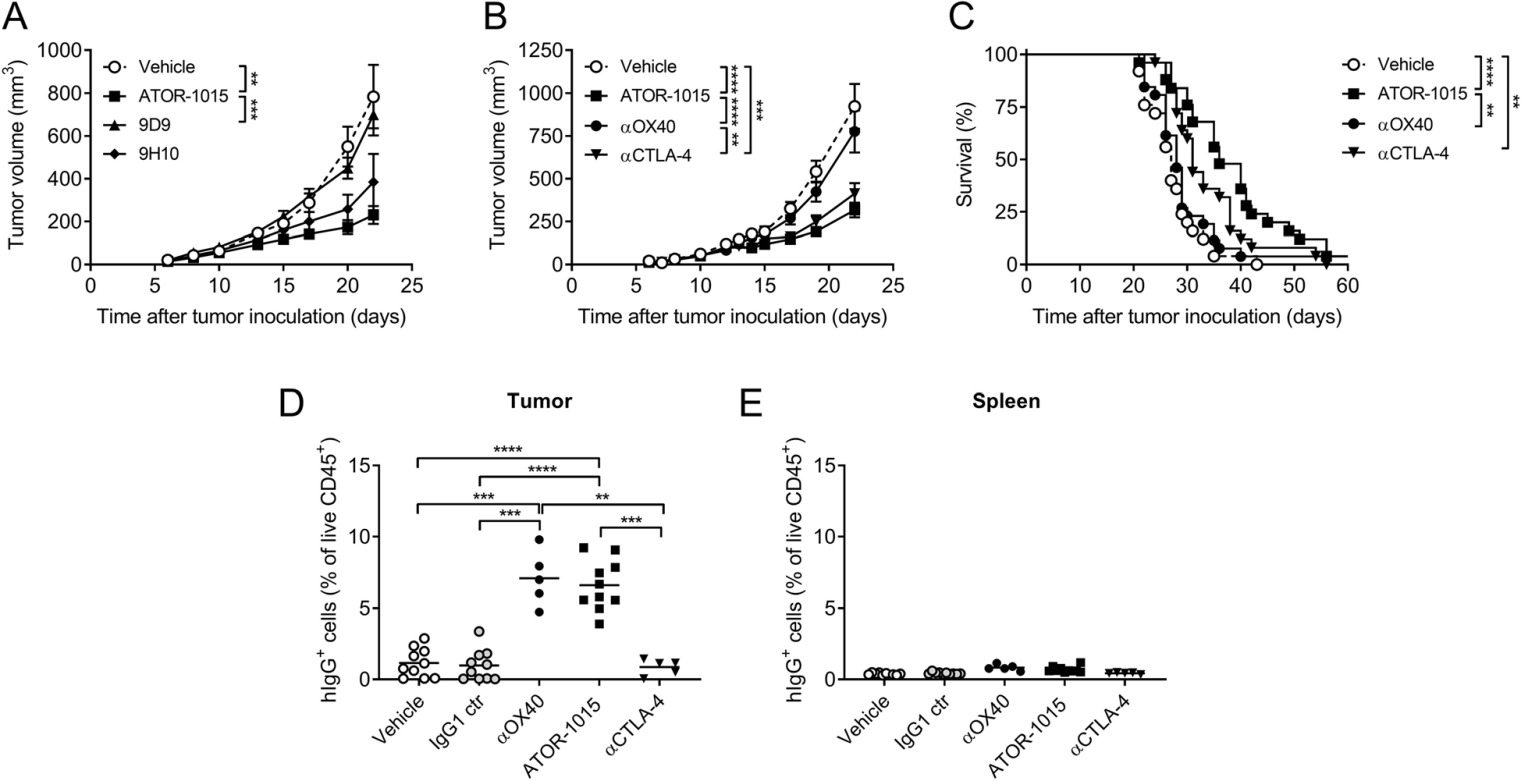
ATOR-1015 localizes to the tumor and induces anti-tumor responses. (**a**) Homozygous hOX40tg mice were inoculated with MC38 cells day 0 and treated ip on days 7, 10 and 13 with antibodies (248 μg for bsAbs or 200 μg for mAbs) as indicated. Tumor volume in mice treated with ATOR-1015, surrogate anti-CTLA-4 antibodies (9D9 and 9H10) or vehicle (n = 7–10). (**b**-**c**) Tumor volume and survival after treatment with ATOR-1015, monotargeting anti-OX40 and anti-CTLA-4 antibodies or vehicle (*n* = 25–26). (**d**, **e**) Mice were treated once with ATOR-1015, monotargeting anti-OX40 and anti-CTLA-4 antibodies, IgG1 control or vehicle on day 17. Twenty-four hours later, tumors and spleens were collected and the level of hIgG^+^ cells was quantified by flow cytometry. Data show the percentage of hIgG^+^ cells out of live CD45^+^ cells (n = 5–10). All data presented as mean ± SEM. Statistical differences were analyzed using Mann-Whitney, two-tailed test, except survival that was analyzed using Kaplan-Meier, Log-Rank (*, p < 0.05; **, p < 0.01; ***, p < 0.001; ****, p < 0.0001). n equals the number of mice

Anti-tumor effects of ATOR-1015 were also demonstrated in the MC38 colon carcinoma model. Homozygous tumor-bearing hOX40tg mice were treated days 10, 13 and 17 with vehicle, ATOR-1015 or the surrogate anti-CTLA-4 antibodies 9D9 (mouse IgG2a) or 9H10 (hamster IgG), the latter being a more effective Treg depleter. ATOR-1015 gave rise to the most pronounced tumor growth inhibition. The response was statistically significant compared to vehicle and 9D9, but not to 9H10 (Fig. 5a). Next, the anti-tumor response of ATOR-1015 was compared to the monotargeting anti-OX40 and anti-CTLA-4 counterparts administered on days 7, 10 and 13. No significant effects were seen with the anti-OX40 antibody, whereas ATOR-1015 and the anti-CTLA-4 antibody induced potent anti-tumor responses (Fig. 5b,c).

ATOR-1015 was designed to induce tumor-directed immune activation by targeting two receptors highly expressed on tumor infiltrating Tregs. Therefore, the ability to bind selectively to tumor-infiltrating T cells was studied. Homozygous mice inoculated with MC38 tumor cells day 0 were treated with vehicle, IgG1 control, monotargeting antibodies or ATOR-1015 once on day 17. Twenty-four hours later, tumors and spleens were collected, and the binding of ATOR-1015 to cells in the tumors and spleens was measured as percentage of hIgG^+^ cells out of the total live CD45^+^ cells. No hIgG^+^ cells were detected in the groups treated with vehicle or IgG1 control. ATOR-1015 and the anti-OX40 antibody were found to selectively bind to the tumor-infiltrating cells, whereas this was not seen with the anti-CTLA-4 antibody. No binding to splenocytes was seen with any of the compounds (Fig. 5d,e).

**Fig. 6:**
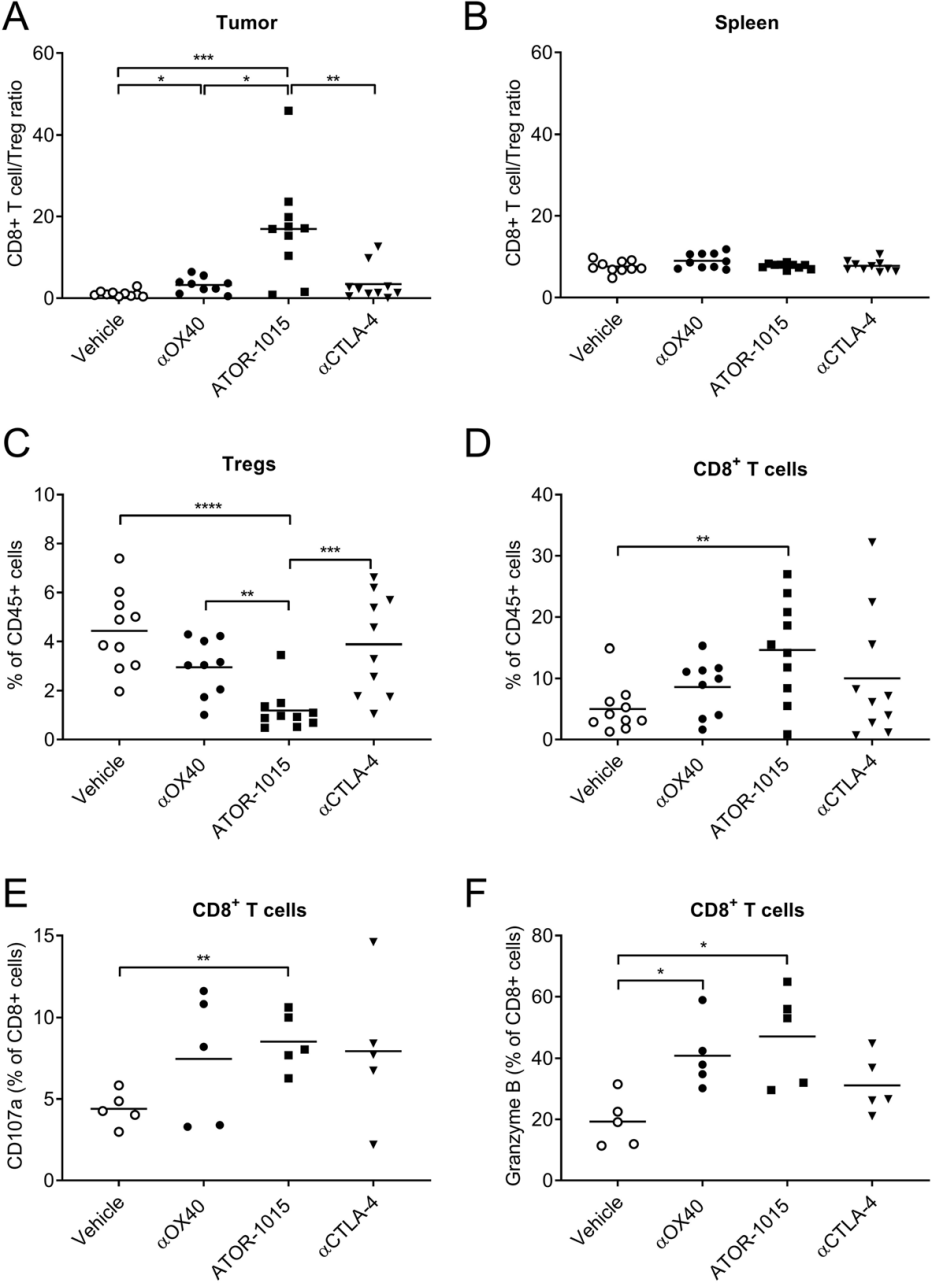
ATOR-1015 depletes Tregs and activates effector T cells in the tumor. Homozygous hOX40tg mice were inoculated with MC38 cells day 0 and treated ip with ATOR-1015, monotargeting anti-CTLA-4 and anti-OX40 antibodies (248 μg for bsAbs or 200 μg for mAbs) or vehicle on days 10, 14 and, 18. Twenty-four hours after the last injection, the tumors and spleens were harvested, and stained for Treg and effector T cell markers. (**a**-**b**) CD8^+^ T cell/Treg ratio in the tumor and spleen. (**c**) Percentage of Tregs (of CD45^+^ cells) in tumors. (**d**) Percentage CD8^+^ T cells (of CD45^+^ cells) in tumors. (**e**-**f**) Expression of CD107a and Granzyme B on CD8^+^ T cells in the tumors. All data presented as mean and each dot represents one animal. Statistical differences were analyzed using Mann-Whitney, two-tailed test, (*, *p* < 0–05; **, *p* < 0.01; ***, *p* < 0.001; ****, *p* < 0.0001)

To further investigate the tumor-directed effects of ATOR-1015, the number and activation status of Tregs and CD8^+^ T cells were investigated. Homozygous mice with MC38 tumors were treated with ATOR-1015, monotargeting antibodies or vehicle on days 10, 14 and 18, and analyzed by flow cytometry 24 h after the last injection. ATOR-1015 significantly increased the CD8^+^ T cell/Treg ratio in the tumor, but not in the spleen (Fig. 6a, b). The effects in the tumor seemed to be a result of both a decrease in Tregs (Fig. 6c), and an increase in CD8^+^ T cells (Fig. 6d). The effects of ATOR-1015 were found to be superior compared to single-agent therapy. Almost identical results were obtained in hOX40tg mice with MB49 tumors (Additional file 5: Figure S3). Furthermore, ATOR-1015 up-regulated the expression of CD107a and Granzyme B on the CD8^+^ T cells, suggesting that they acquire a cytotoxic phenotype (Fig. 6e, f).

### ATOR-1015 enhances the effect of anti-PD-1 treatment

**Fig. 7:**
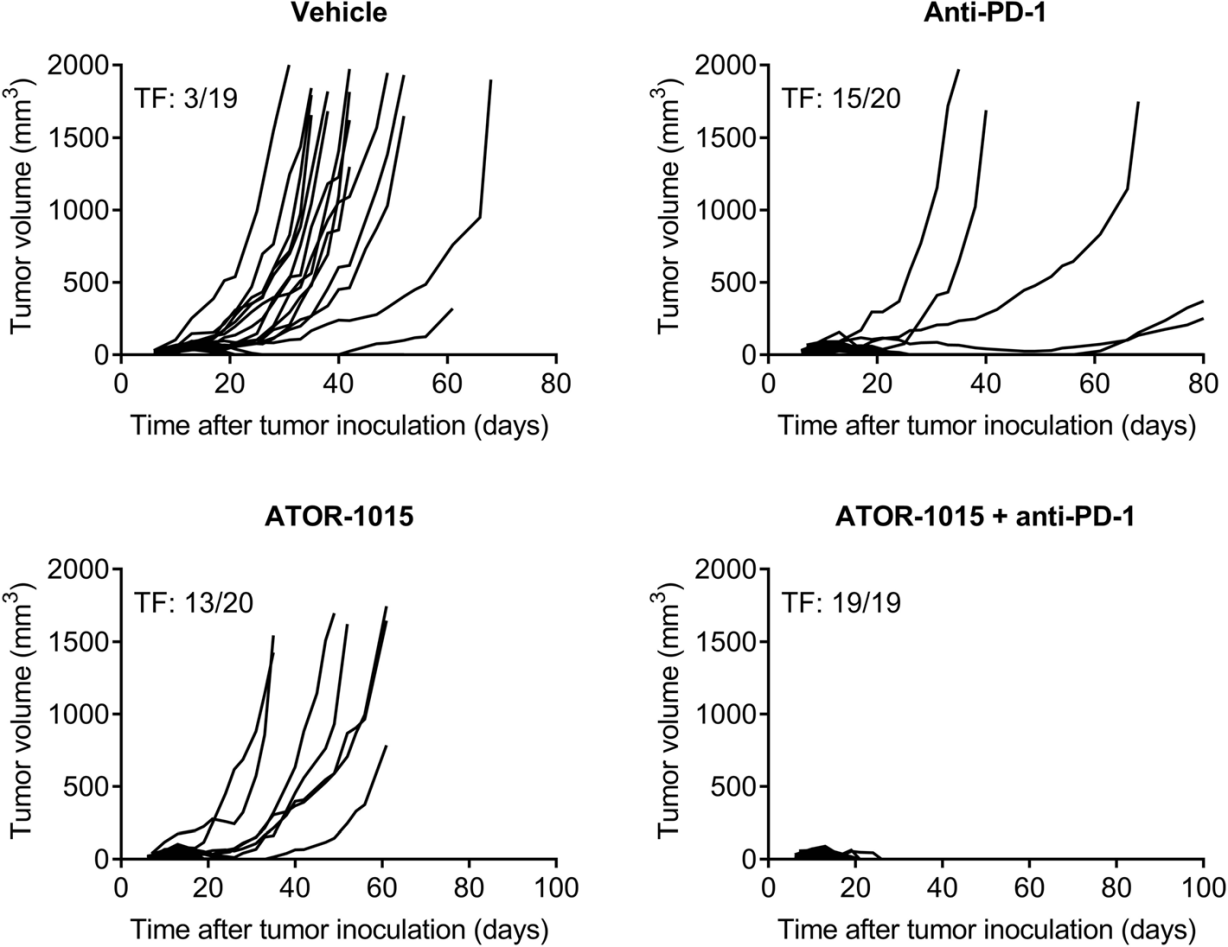
ATOR-1015 improves the effect of anti-PD-1 treatment. Female heterozygous hOX40tg mice inoculated sc with MB49 tumor cells day 0 were treated ip with vehicle, 248 μg ATOR-1015, and/or 250 μg anti-PD-1 (RPM1–14) on days 7, 10 and 13. Tumor volume was measured three times per week. The number of tumor free (TF) mice in each group is indicated

The ability of ATOR-1015 to improve the effect of anti-PD-1 antibodies was tested in hOX40tg mice with bladder or colon carcinoma. Mice were injected sc with tumor cells on day 0 and treated ip with 248 μg ATOR-1015 and/or 250 μg anti-PD-1 on days 7, 10 and 13. In heterozygous hOX40tg mice with MB49 cancer, monotherapy with ATOR-1015 or anti-PD-1 induced potent anti-tumor responses and complete remission in a large fraction of the mice. The combination of ATOR-1015 and anti-PD-1 cured all mice from MB49 cancer (Fig. 7). Similarly, in mice with MC38 and CT26 tumors, ATOR-1015 significantly enhanced the activity of anti-PD-1, both in terms of tumor growth inhibition and survival (Additional file 6: Figure S4).

## Discussion

ATOR-1015 is a human CTLA-4 x OX40 bispecific IgG1 antibody. It has a dual mode of action, including depletion of Tregs and activation of effector T cells in the tumor. Both CTLA-4 and OX40 are highly up-regulated on tumor-infiltrating Tregs in several types of cancer. By targeting both receptors simultaneously, the functional activity of ATOR-1015 is directed to the tumor area. Herein, we demonstrate that ATOR-1015 treatment induces anti-tumor responses and improves survival in several syngeneic tumor models. The response is tumor-specific and provides long-term immunological memory in cured mice. Moreover, we show in mouse tumor models that ATOR-1015 localizes to the tumor where it reduces the frequency of Tregs and increases the number and activation of CD8^+^ T cells. Lastly, ATOR-1015 enhances the anti-tumor response to anti-PD-1 treatment.

ATOR-1015 requires engagement with CTLA-4 and/or Fcγ receptors to induce OX40 receptor clustering and T-cell activation. This indicates that ATOR-1015 is less likely to activate T cells in the periphery where expression of CTLA-4 and OX40 is low. Instead, ATOR-1015 activates T cells in the tumor where target expression and density of Fcγ receptor-expressing cells are high, but not in the spleen. This is in line with previous studies showing that most agonistic TNFR antibodies depend on crosslinking for induction of receptor superclustering to achieve a good agonistic effect [23, 27, 28], and that CTLA-4-mediated T-cell signaling and function is improved upon Fc-FcγR engagement [29].

The ability of ATOR-1015 to induce depletion of Tregs has been demonstrated both in vitro and in vivo. Using an ADCC assay with primary Tregs and NK cells, ATOR-1015 induces target cell lysis to a similar extent as the combination of anti-OX40 and anti-CTLA-4 antibodies. The OX40 expression on the Tregs was very high whereas CTLA-4 was barely detectable, indicating that the responses seen in vitro mainly are mediated by OX40. However, in the ADCC reporter assay with Tregs or target-expressing CHO cells, ATOR-1015 is superior to the combination of monotargeting antibodies. The reason for this discrepancy between assays is not known, but it might be that reporter assays that measures FcγRIIIa engagement are more sensitive to avidity effects, whereas the NK cell-mediated lysis of Tregs is more dependent on target receptor density. Intratumoral expression of the NK cell marker CD56 has been shown to correlate with clinical response to ipilimumab in patients with melanoma, suggesting that anti-CTLA-4-induced Treg depletion is mediated at least in part by NK cells [30]. Non-classical monocytes in the TME also express FcγRIIIa, and they have been reported to partake in the Treg depletion in cancer patients treated with ipilimumab [11]. Further, an increase in the CD8^+^ T cell/Treg ratio in the tumor is demonstrated in mice bearing MC38 or MB49 tumors following ATOR-1015 treatment, whereas the ratio is unchanged in the spleen, indicating that there are no systemic effects. No change in the CD8^+^ T cell/Treg ratio was seen with the monotargeting anti-CTLA-4 antibody. Several studies have demonstrated that anti-mouse CTLA-4 antibodies induce depletion of intratumoral Tregs [6, 31–33], but there are also studies where this could not be detected [21, 29, 34]. Increased intratumoral CD8^+^ T cell/Treg ratio has been associated with therapeutic response both in pre-clinical studies in mice [8, 32, 35], and in cancer patients [36, 37]. In the present study, the observed increased ratio with ATOR-1015 is a result of both Treg depletion and expansion or infiltration of CD8^+^ effector T cells compared to monospecific anti-CTLA-4 treatment. Dual targeting of CTLA-4 and OX40 clearly improves the Treg depletion in these in vivo models. Moreover, targeting of OX40 increase the amount of ATOR-1015 detected in the tumor area.

It is still debated whether CTLA-4 blocking antibodies deplete intratumoral Tregs in cancer patients. While several studies have demonstrated a reduction of Tregs in tumor tissues in patients treated with ipilimumab [36, 38], a recent study by Sharma et al. reported an increased rather than a reduced density of Foxp3^+^ Tregs in stage-matched tumors samples treated with ipilimumab and paired tumors treated with tremelimumab [12]. However, as suggested by others there are limitations with this study, including timing of biopsies and difficulties in identifying membrane-CTLA-4^+^ Tregs by immunohistochemistry as it is also highly expressed within the cytoplasm of CD4^+^ T cells [13, 39]. Moreover, depletion of intratumoral Tregs is proposed to occur selectively in patients responding to CTLA-4 inhibitors [11], and yet, the study lacks information whether the patients showed clinical response or progression. It has been proposed that the development of next generation anti-CTLA-4 antibodies with enhanced ADCC will drive more potent and durable responses [12, 13]. By targeting both OX40 and CTLA-4, ATOR-1015 induces an enhanced and tumor-directed Treg depletion, which may translate into a better clinical response both in terms of efficacy and safety. However, evaluating toxicity to anti-CTLA-4 antibodies in mice is difficult as the immune-related adverse events commonly observed in patients rarely occur in mice [31]. Nevertheless, signs of immune-related toxicity in the treated mice have not been observed, i.e. no skin, hair or eye changes or wasting syndrome.

The anti-tumor responses of ATOR-1015 were studied in a novel hOX40tg mouse model, enabling studies of both OX40- and CTLA-4-mediated effects. ATOR-1015 shows potent anti-tumor activity and improved survival in MB49 and MC38. Interestingly, the overall anti-tumor effect of ATOR-1015 in MB49 was higher in heterozygous than in homozygous hOX40tg mice, despite lower expression levels of OX40. Heterozygotes were obtained by breeding male homozygotes (C57BL/6) with female BALB/c mice. Thus, the differences in anti-tumor activity are likely explained by the different genetic background and immunological variation between the strains. It is well known that C57BL/6 and BALB/c are different in e.g. Th1/Th2 polarization, MHC haplotype and NK cell phenotype [40, 41]. In addition, anti-tumor effects are demonstrated in CT26 colon carcinoma and PANC02 pancreas cancer models (Additional file 7: Figure S5 and Additional file 8: Figure S6). The resulting immune response is tumor-specific and provides long-term immunological memory. However, no effects on tumor growth or survival were observed in the B16.F10 melanoma or A20 B cell lymphoma models (Additional file 9: Figure S7). The lack of response in B16.F10 probably relates to its invasive and low immunogenic nature. It is well known that IO drugs administered as single agents, including CTLA-4, generally are poor at inducing anti-tumor responses in this model [42–44]. In A20, analysis of infiltrating lymphocytes revealed very few CD4^+^, CD8^+^ and Treg cells compared to what was seen in MB49 and MC38, which may explain the lack of effect. Thus, our data supports clinical testing of ATOR-1015 in cancer patients, particularly in patients with T-cell infiltrated tumors such as melanoma, NSCLC and RCC, based on the clinical data from studies with ipilimumab.

The combination of anti-OX40 and anti-CTLA-4 antibodies has been tested in some tumor models, and the resulting anti-tumor effect was found to be *on par* with ATOR-1015 (data not shown). However, ATOR-1015 was designed as a next generation CTLA-4 targeting antibody, with the aim of having a more favourable safety profile and better efficacy than current anti-CTLA-4 antibodies due to its tumor-directed activity. ATOR-1015 was developed for future combination with anti-PD-(L)1 in the clinic, and will be benchmarked to other anti-CTLA-4 plus anti-PD-(L)1 regimens in terms of safety and efficacy.

Lastly, it is demonstrated that ATOR-1015 enhances the effect of anti-PD-1 treatment in mice with bladder and colon cancer. Combination therapies with antibodies targeting CTLA-4 and the PD-1/PD-L1 axis are approved in several indications, and there are more than 250 ongoing trials [1]. While the combination is very effective in some indications, the rate of adverse events doubles compared to single-agent therapy, with treatment-related adverse events of grade 3–4 in > 50% of the patients [45–47]. This has limited the number of patients that receive and tolerate the treatment and led to the use of sub-maximally effective doses for combinations with PD-1. CTLA-4 therapies with optimized clinical benefit to adverse event ratio would therefore enable more effective treatment, move combination therapy into frontline, and increase the potential for neoadjuvant combination therapy in more indications.

## Conclusions

ATOR-1015 is a next generation CTLA-4 targeting antibody with enhanced immune activation and tumor-directed activity, which may translate into improved clinical efficacy and reduced toxicity compared to anti-CTLA-4 monotherapy. The potentially improved benefit-risk profile of ATOR-1015 due to its tumor-directed activity may enable treatment of larger patient populations either as a standalone treatment or in combination with anti-PD-(L)1 in a wide spectrum of indications. The pre-clinical data presented herein support clinical development of ATOR-1015. GLP safety studies have been completed and a first-in-human trial has recently started (NCT03782467).

## Additional files

Additional file 1: Supplementary Method (DOCX 20 kb)

Additional file 2: **Figure S1.** Characterization of the CTLA-4 binding domain. (DOCX 59 kb)

Additional file 3: **Table S1.** Kinetic constants for ATOR-1015 binding to OX40 and CTLA-4 in Biacore. (DOCX 15 kb)

Additional file 4: **Figure S2**. Characterization of the hOX40tg mouse model. (DOCX 165 kb)

Additional file 5: **Figure S3**. ATOR-1015 depletes Tregs and activates effector T cells in the tumor. (DOCX 186 kb)

Additional file 6: **Figure S4**. ATOR-1015 improves the effect of PD-1 blocking antibodies. (DOCX 206 kb)

Additional file 7: **Figure S5**. Anti-tumor effect of ATOR-1015 in hOX40tg mice bearing CT26 colon carcinoma. (DOCX 100 kb)

Additional file 8: **Figure S6**. Anti-tumor effect of ATOR-1015 in hOX40tg mice bearing PANC02 pancreas cancer. (DOCX 103 kb)

Additional file 9: **Figure S7**. Non-responsive models. (DOCX 117 kb)

## Abbreviations

ADCC: Antibody-dependent cell-mediated cytotoxicity
ADCP: antibody-dependent cellular phagocytosis
bsAb: bispecific antibody
CHO: Chinese hamster ovary
FcγR: Fcγ receptor
GITR: Glucocorticoid-induced TNFR-related protein
HEK: Human embryonic kidney
IO: Immuno-oncology
ip: Intraperitoneal
LDH: Lactate dehydrogenase
MFI: Mean fluorescence intensity
NFAT: Nuclear factor of activated T cells
NSCLC: Non-small cell lung cancer
OX40L: OX40 ligand
PBMC: Peripheral blood mononuclear cells
PD-1: Programmed cell death protein
PD-L1: Programmed death-ligand 1
RCC: Renal cell carcinoma
sc: subcutaneous
TME: Tumor microenvironment
TNFR: Tumor necrosis family receptor
Treg: T regulatory cell
wt: wildtype

## References

[1] Tang J, Shalabi A, Hubbard-Lucey VM (2018). Comprehensive analysis of the clinical immuno-oncology landscape. Ann Oncol.

[2] Wei SC, Duffy CR, Allison JP (2018). Fundamental mechanisms of immune checkpoint blockade therapy. Cancer Discov.

[3] Marin-Acevedo JA, Dholaria B, Soyano AE, Knutson KL, Chumsri S, Lou Y (2018). Next generation of immune checkpoint therapy in cancer: new developments and challenges. J Hematol Oncol.

[4] Sledzinska A, Menger L, Bergerhoff K, Peggs KS, Quezada SA (2015). Negative immune checkpoints on T lymphocytes and their relevance to cancer immunotherapy. Mol Oncol.

[5] Jago CB, Yates J, Camara NO, Lechler RI, Lombardi G (2004). Differential expression of CTLA-4 among T cell subsets. Clin Exp Immunol.

[6] Arce Vargas F, Furness AJS, Litchfield K, Joshi K, Rosenthal R, Ghorani E (2018). Fc effector function contributes to the activity of human anti-CTLA-4 antibodies. Cancer Cell.

[7] Montler R, Bell RB, Thalhofer C, Leidner R, Feng Z, Fox BA (2016). OX40, PD-1 and CTLA-4 are selectively expressed on tumor-infiltrating T cells in head and neck cancer. Clin Transl Immunology.

[8] Du X, Tang F, Liu M, Su J, Zhang Y, Wu W (2018). A reappraisal of CTLA-4 checkpoint blockade in cancer immunotherapy. Cell Res.

[9] Gombos RB, Gonzalez A, Manrique M, Chand D, Savitsky D, Morin B (2018). Toxicological and pharmacological assessment of AGEN1884, a novel human IgG1 anti-CTLA-4 antibody. PLoS One.

[10] Ingram JR, Blomberg OS, Rashidian M, Ali L, Garforth S, Fedorov E (2018). Anti-CTLA-4 therapy requires an fc domain for efficacy. Proc Natl Acad Sci U S A.

[11] Romano E, Kusio-Kobialka M, Foukas PG, Baumgaertner P, Meyer C, Ballabeni P (2015). Ipilimumab-dependent cell-mediated cytotoxicity of regulatory T cells ex vivo by nonclassical monocytes in melanoma patients. Proc Natl Acad Sci.

[12] Sharma Anu, Subudhi Sumit K., Blando Jorge, Scutti Jorge, Vence Luis, Wargo Jennifer, Allison James P., Ribas Antoni, Sharma Padmanee (2018). Anti-CTLA-4 Immunotherapy Does Not Deplete FOXP3+ Regulatory T Cells (Tregs) in Human Cancers. Clinical Cancer Research.

[13] Quezada Sergio A., Peggs Karl S. (2018). Lost in Translation: Deciphering the Mechanism of Action of Anti-human CTLA-4. Clinical Cancer Research.

[14] Schadendorf D, Hodi FS, Robert C, Weber JS, Margolin K, Hamid O (2015). Pooled analysis of long-term survival data from phase II and phase III trials of Ipilimumab in Unresectable or metastatic melanoma. J Clin Oncol.

[15] Eroglu Z, Kim DW, Wang X, Camacho LH, Chmielowski B, Seja E (2015). Long term survival with cytotoxic T lymphocyte-associated antigen 4 blockade using tremelimumab. Eur J Cancer.

[16] Hodi FS (2010). Overcoming immunological tolerance to melanoma: Targeting CTLA-4. Asia Pac J Clin Oncol.

[17] Bertrand A, Kostine M, Barnetche T, Truchetet ME, Schaeverbeke T (2015). Immune related adverse events associated with anti-CTLA-4 antibodies: systematic review and meta-analysis. BMC Med.

[18] Ellmark P, Mangsbo SM, Furebring C, Norlen P, Totterman TH (2017). Tumor-directed immunotherapy can generate tumor-specific T cell responses through localized co-stimulation. Cancer Immunol Immunother.

[19] Vetto JT, Lum S, Morris A, Sicotte M, Davis J, Lemon M (1997). Presence of the T-cell activation marker OX-40 on tumor infiltrating lymphocytes and draining lymph node cells from patients with melanoma and head and neck cancers. Am J Surg.

[20] Galon J, Bruni D. Approaches to treat immune hot, altered and cold tumours with combination immunotherapies. Nat Rev Drug Discov. 2019. 10.1038/s41573-018-0007-y 30610226

[21] Redmond WL, Linch SN, Kasiewicz MJ (2014). Combined targeting of costimulatory (OX40) and coinhibitory (CTLA-4) pathways elicits potent effector T cells capable of driving robust antitumor immunity. Cancer Immunol Res..

[22] Emerson DA, Redmond WL (2018). Overcoming tumor-induced immune suppression: from relieving inhibition to providing Costimulation with T cell agonists. BioDrugs..

[23] Mayes PA, Hance KW, Hoos A (2018). The promise and challenges of immune agonist antibody development in cancer. Nat Rev Drug Discov.

[24] Glisson BS, Leidner R, Ferris RL, Powderly J, Rizvi N, Norton JD, Burton J, Lanasa MC, Patel SP (2016). Phase 1 study of MEDI0562, a humanized OX40 agonist monoclonal antibody (mAb), in adult patients (pts) with advanced solid tumors. Ann Oncol.

[25] Jie HB, Gildener-Leapman N, Li J, Srivastava RM, Gibson SP, Whiteside TL (2013). Intratumoral regulatory T cells upregulate immunosuppressive molecules in head and neck cancer patients. Br J Cancer.

[26] Syed Khaja AS, Toor SM, El Salhat H, Ali BR, Elkord E (2017). Intratumoral FoxP3(+)Helios(+) regulatory T cells upregulating immunosuppressive molecules are expanded in human colorectal Cancer. Front Immunol.

[27] Stewart R, Hammond SA, Oberst M, Wilkinson RW (2014). The role of fc gamma receptors in the activity of immunomodulatory antibodies for cancer. Journal for ImmunoTherapy of Cancer.

[28] Waight JD, Gombos RB, Wilson NS. Harnessing co-stimulatory TNF receptors for cancer immunotherapy: current approaches and future opportunities. Hum Antibodies. 2017. 10.3233/HAB-160308 28085016

[29] Waight JD, Chand D, Dietrich S, Gombos R, Horn T, Gonzalez AM (2018). Selective FcgammaR co-engagement on APCs modulates the activity of therapeutic antibodies targeting T cell antigens. Cancer Cell.

[30] Stone E, O'Brien EM, Sanseviero E, Karras J, Shabaneh T, Aksoy BA, et al., Editors. Anti-CTLA4 activation of intratumoral NK cells may contribute to intratumoral Treg depletion. SITC'18, Washington DC; 9-11 November 2018. 6(suppl 1), Abstract O40.

[31] Sandin LC, Eriksson F, Ellmark P, Loskog AS, Totterman TH, Mangsbo SM (2014). Local CTLA4 blockade effectively restrains experimental pancreatic adenocarcinoma growth in vivo. Oncoimmunology..

[32] Selby M. J., Engelhardt J. J., Quigley M., Henning K. A., Chen T., Srinivasan M., Korman A. J. (2013). Anti-CTLA-4 Antibodies of IgG2a Isotype Enhance Antitumor Activity through Reduction of Intratumoral Regulatory T Cells. Cancer Immunology Research.

[33] Simpson TR, Li F, Montalvo-Ortiz W, Sepulveda MA, Bergerhoff K, Arce F (2013). Fc-dependent depletion of tumor-infiltrating regulatory T cells co-defines the efficacy of anti-CTLA-4 therapy against melanoma. J Exp Med.

[34] Mangsbo SM, Sandin LC, Anger K, Korman AJ, Loskog A, Totterman TH (2010). Enhanced tumor eradication by combining CTLA-4 or PD-1 blockade with CpG therapy. J Immunother.

[35] Quezada SA, Peggs KS, Curran MA, Allison JP (2006). CTLA4 blockade and GM-CSF combination immunotherapy alters the intratumor balance of effector and regulatory T cells. J Clin Invest.

[36] Hodi FS, Butler M, Oble DA, Seiden MV, Haluska FG, Kruse A (2008). Immunologic and clinical effects of antibody blockade of cytotoxic T lymphocyte-associated antigen 4 in previously vaccinated cancer patients. Proc Natl Acad Sci U S A.

[37] Sato E, Olson SH, Ahn J, Bundy B, Nishikawa H, Qian F (2005). Intraepithelial CD8+ tumor-infiltrating lymphocytes and a high CD8+/regulatory T cell ratio are associated with favorable prognosis in ovarian cancer. Proc Natl Acad Sci U S A.

[38] Liakou CI, Kamat A, Tang DN, Chen H, Sun J, Troncoso P (2008). CTLA-4 blockade increases IFNgamma-producing CD4+ICOShi cells to shift the ratio of effector to regulatory T cells in cancer patients. Proc Natl Acad Sci U S A.

[39] Ferrara R, Susini S, Marabelle A. Anti-CTLA-4 immunotherapy does not deplete FOXP3+ regulatory T cells (Tregs) in human cancers-letter. Clin Cancer Res. 2018. 10.1158/1078-0432.CCR-18-3740 30514779

[40] Sellers RS, Clifford CB, Treuting PM, Brayton C (2012). Immunological variation between inbred laboratory mouse strains: points to consider in phenotyping genetically immunomodified mice. Vet Pathol.

[41] Fiserova A, Richter J, Capkova K, Bieblova J, Mikyskova R, Reinis M (2016). Resistance of novel mouse strains different in MHC class I and the NKC domain to the development of experimental tumors. Int J Oncol.

[42] van Elsas A, Hurwitz AA, Allison JP (1999). Combination immunotherapy of B16 melanoma using anti-cytotoxic T lymphocyte-associated antigen 4 (CTLA-4) and granulocyte/macrophage colony-stimulating factor (GM-CSF)-producing vaccines induces rejection of subcutaneous and metastatic tumors accompanied by autoimmune depigmentation. J Exp Med.

[43] Xu D, Gu P, Pan PY, Li Q, Sato AI, Chen SH (2004). NK and CD8+ T cell-mediated eradication of poorly immunogenic B16-F10 melanoma by the combined action of IL-12 gene therapy and 4-1BB costimulation. Int J Cancer.

[44] Selby MJ, Engelhardt JJ, Johnston RJ, Lu LS, Han M, Thudium K (2016). Preclinical development of Ipilimumab and Nivolumab combination immunotherapy: mouse tumor models, in vitro functional studies, and Cynomolgus macaque toxicology. PLoS One.

[45] Wolchok JD, Chiarion-Sileni V, Gonzalez R, Rutkowski P, Grob JJ, Cowey CL, et al. Overall Survival with Combined Nivolumab and Ipilimumab in Advanced Melanoma. N Engl J Med. 2017. 10.1056/NEJMoa1709684 PMC5706778 28889792

[46] Tawbi HA, Forsyth PA, Algazi A, Hamid O, Hodi FS, Moschos SJ (2018). Combined Nivolumab and Ipilimumab in melanoma metastatic to the brain. N Engl J Med.

[47] Postow MA, Chesney J, Pavlick AC, Robert C, Grossmann K, McDermott D (2015). Nivolumab and ipilimumab versus ipilimumab in untreated melanoma. N Engl J Med.

