# Supplementary material for: The CTLA-4 x OX40 bispecific antibody ATOR-1015 induces anti-tumor effects through tumor-directed immune activation
Source: J Immunother Cancer. 2019 Apr 11;7:103. doi: 10.1186/s40425-019-0570-8 (PMC6458634; doi:10.1186/s40425-019-0570-8)
Supplement: Supplementary file 1 — Supplementary Method (DOCX 20 kb) [file 40425_2019_570_MOESM1_ESM.docx]

Additional file 1: Supplementary Method

*Generation of the CTLA-4 binding part of ATOR-1015*

Libraries were generated based on the IgGV domain of CD86 (aa 24-134). The libraries were designed to include mutations in surface exposed residues in the beta sheets containing the binding epitope to CTLA-4. Alternatively, libraries were generated by random mutations (error prone PCR). The libraries were expressed in a M13 phagemid format. Selections were performed using biotinylated human CTLA-4 as target and catching CTLA-4 bound phages on magnetic beads. Soluble (non-biotinylated) CD28 (200-300 nM) was used to de-select binders with improved affinity for CD28. A 60°C incubation step was introduced to select for binders with high thermostability. Following phage selection, binders were screened for improved binding to CTLA-4 and improved CTLA-4/CD28 binding ratio. Identified sequences were then recombined using the FIND^®^ technology to generate an additional library. The FIND^®^ recombined library was subjected to new rounds of selection and the obtained binders were screened for improved binding and specificity. The top ten clones were transferred to a bispecific format and screened for affinity to target, specificity, developability and ability to block the CTLA-4/CD86 interaction. The final clone, containing 5 mutations in the binding epitope had a ~100-fold increase in affinity as determined by the IC_50_ value compared to the starting sequence.

*Generation of the OX40 agonist antibody part of ATOR-1015*

Phage display selections were performed from the ALLIGATOR-GOLD^®^ human antibody library. Selections (5 rounds) were performed using in-house biotinylated hOX40-Fc in solution. The non-biotinylated protein was purchased from R&D Systems. Beriglobin and an irrelevant His-tagged protein were used as non-targets in excess in all selection rounds. Washes were performed with increased stringency during the selections. Prior to each selection round, the phage stocks were pre-selected against streptavidin coated Dynabeads M-280 (Life Technologies). After multiple rounds of selections, 2300 clones were evaluated in high-throughput screening ELISA. Specificity was evaluated by assessing the ability to bind to non-target proteins: Streptavidin, BSA, Transthyretin, Transferrin, hGITR-Fc, and Ubiquitin-His, hEGFr-His. Clones with unique sequences were identified and evaluated for functional activity in full IgG format. Seven OX40 antibodies that met the selection criteria were further evaluated for specificity, epitope binding, developability, affinity and ability to stimulate OX40 on T cells. A crosslinking-dependent OX40 specific antibody was selected.

*Binding affinity determination*

Either CTLA4-Fc (Fitzgerald) or human OX40 (R&D Systems) was immobilized to the Biacore™ sensor chip, CM5, using conventional amine coupling. The CD86 mutant molecules, ATOR-1015 and controls were serially diluted and analyzed for binding in HBS-P (GE Healthcare) at a flow rate of 30 µl/ml. The association was followed for 3 minutes and the dissociation for 20 or 10 minutes. Regeneration was performed twice using 5 mM NaOH for 30 seconds. The kinetic parameters and the affinity constants were calculated using BIAevaluation 4.1 software.

*OX40 domain mapping*

Genes of OX40 human/mouse chimeras were synthesized (GenScript). The different chimeras were designed by exchanging domains or modules of the human OX40 with corresponding mouse OX40. The chimeras were designed based on evaluation of the human and mouse sequences and 3D investigation of human OX40. The constructs were cloned into pcDNA3.1 vector (Invitrogen). The mouse/human chimeras were transiently transfected into FreeStyle 293-F cells (Invitrogen), incubated 48 h in FreeStyle 293 expression medium (Invitrogen) at 37°C, 8% CO_2_ and 135 rpm. The transfected cells were incubated with ATOR-1015, human OX40L, mouse OX40L (both R&D Systems) and controls for 30 min at 4°C and then detected with anti-hIgG-PE (Jackson Immunoresearch) for 30 min at 4°C. Cells were analyzed with FACS Verse (BD Biosciences). Binding to the different chimeric constructs were calculated as relative MFI compared to the binding of the isotype control, and the binding domain identified based on binding pattern.
