# Supplementary material for: The CTLA-4 x OX40 bispecific antibody ATOR-1015 induces anti-tumor effects through tumor-directed immune activation
Source: J Immunother Cancer. 2019 Apr 11;7:103. doi: 10.1186/s40425-019-0570-8 (PMC6458634; doi:10.1186/s40425-019-0570-8)
Supplement: Supplementary file 2 — Figure S1. Characterization of the CTLA-4 binding domain. (DOCX 59 kb) [file 40425_2019_570_MOESM2_ESM.docx]

Additional file 2: Figure S1


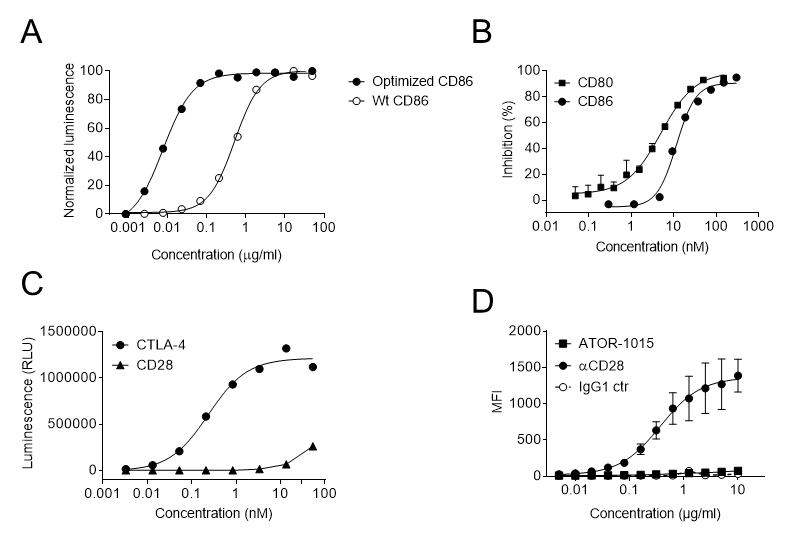


Figure S1. Characterization of the CTLA-4 binding domain. (A) Improved binding to CTLA-4 of the CTLA-4 binding domain of ATOR-1015 compared to wildtype CD86. Binding determined using ELISA and presented as normalized response. (B) ATOR-1015 dose-dependently blocks CTLA-4 from interacting with CD80 and CD86 in a CTLA-4 inhibition assay using CHO cells transfected to express CTLA-4. (C) ATOR-1015 binding to CTLA-4 and CD28 determined by ELISA. Data presented as luminescence in relative light units (RLU). (D) ATOR-1015 does not bind to CD28 on CD3^+^ T cells from PBMC. ATOR-1015, a commercial anti-CD28 antibody and IgG1 control were labelled with AF647. Cells were stained with labelled antibodies and PECy7-labelled anti-CD3, and binding to CD3^+^ T cells was analyzed by flow cytometry. Data presented as mean fluorescence intensity (MFI) (n=4 donors).
