# Supplementary material for: The CTLA-4 x OX40 bispecific antibody ATOR-1015 induces anti-tumor effects through tumor-directed immune activation
Source: J Immunother Cancer. 2019 Apr 11;7:103. doi: 10.1186/s40425-019-0570-8 (PMC6458634; doi:10.1186/s40425-019-0570-8)
Supplement: Supplementary file 3 — Table S1. Kinetic constants for ATOR-1015 binding to OX40 and CTLA-4 in Biacore. (DOCX 15 kb) [file 40425_2019_570_MOESM3_ESM.docx]

Additional file 3: Table S1

**Table S1.** Kinetic constants for ATOR-1015 binding to OX40 and CTLA-4 in Biacore

|  | **ka (1/Ms)** | **kd (1/s)** | **KD (nM)** |
| --- | --- | --- | --- |
| OX40 | 7.0E+04 | 1.1E-04 | 1.6 |
| CTLA-4 | 1.0E+06 | 3.2E-03 | 3.0 |
