# Supplementary material for: The CTLA-4 x OX40 bispecific antibody ATOR-1015 induces anti-tumor effects through tumor-directed immune activation
Source: J Immunother Cancer. 2019 Apr 11;7:103. doi: 10.1186/s40425-019-0570-8 (PMC6458634; doi:10.1186/s40425-019-0570-8)
Supplement: Supplementary file 4 — Figure S2. Characterization of the hOX40tg mouse model. (DOCX 165 kb) [file 40425_2019_570_MOESM4_ESM.docx]

Additional file 4: Figure S2


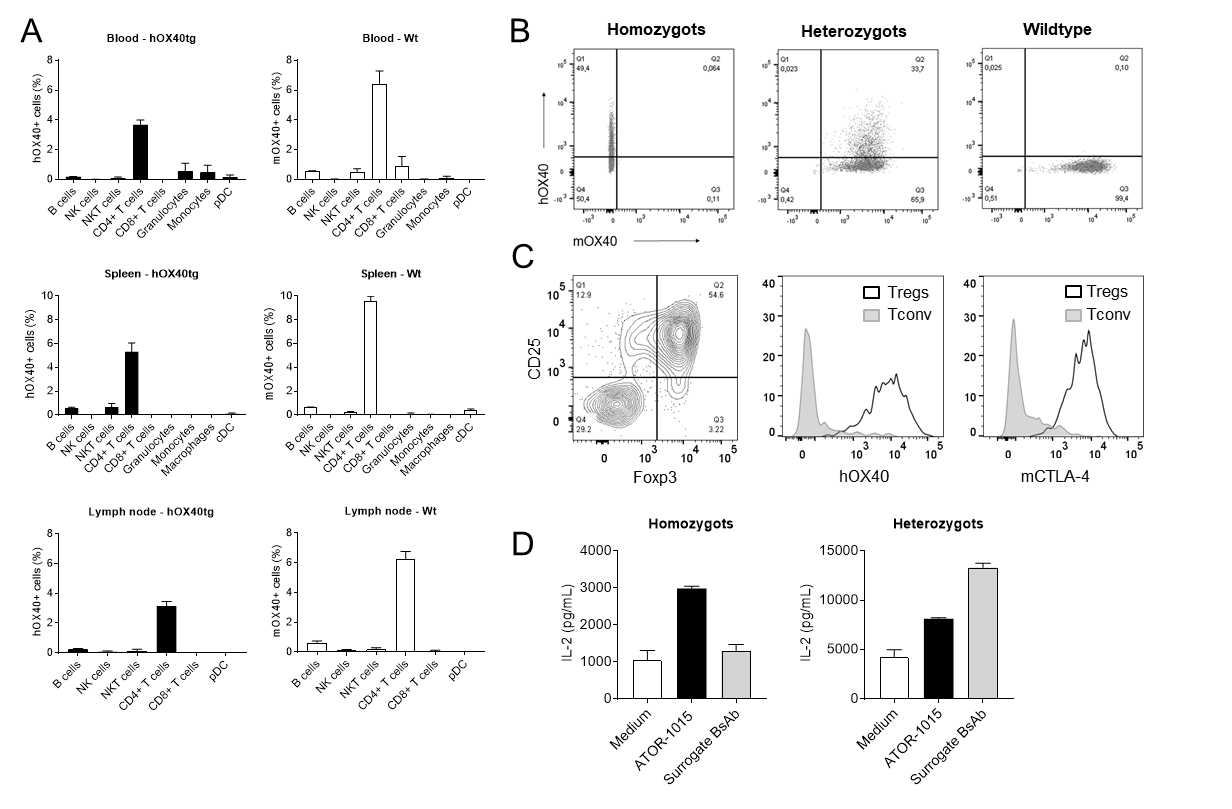


**Figure S2. Characterization of the hOX40tg mouse model.** **(A)** Expression of hOX40 and mOX40 on different cell populations from freshly obtained peripheral blood, spleen and lymph nodes from hOX40tg mice and wildtype (wt) mice (n=5). **(B)** Expression of hOX40 and mOX40 on splenic CD4^+^ T cells from homozygous hOX40tg, heterozygous hOX40tg and wt mice after activation for 48 h with anti-CD3/CD28. **(C)** Expression of hOX40 and mCTLA-4 on conventional T cells (CD4^+^ CD25^-^ Foxp3^-^) and Tregs (CD4^+^ CD25^+^ Foxp3^+^) from tumors (MC38) obtained from homozygous hOX40tg mice. **(D)** CD4^+^ splenocytes from hOX40tg were activated to produce IL-2 in response to ATOR-1015 or a surrogate (mOX40 x CTLA-4) antibody in the presence of suboptimal anti-CD3 and CTLA-4 crosslinking. Data presented as mean ± SEM (n=2). n equals the number of mice.
