# Supplementary material for: The CTLA-4 x OX40 bispecific antibody ATOR-1015 induces anti-tumor effects through tumor-directed immune activation
Source: J Immunother Cancer. 2019 Apr 11;7:103. doi: 10.1186/s40425-019-0570-8 (PMC6458634; doi:10.1186/s40425-019-0570-8)
Supplement: Supplementary file 5 — Figure S3. ATOR-1015 depletes Tregs and activates effector T cells in the tumor. (DOCX 186 kb) [file 40425_2019_570_MOESM5_ESM.docx]

Additional file 5: Figure S3


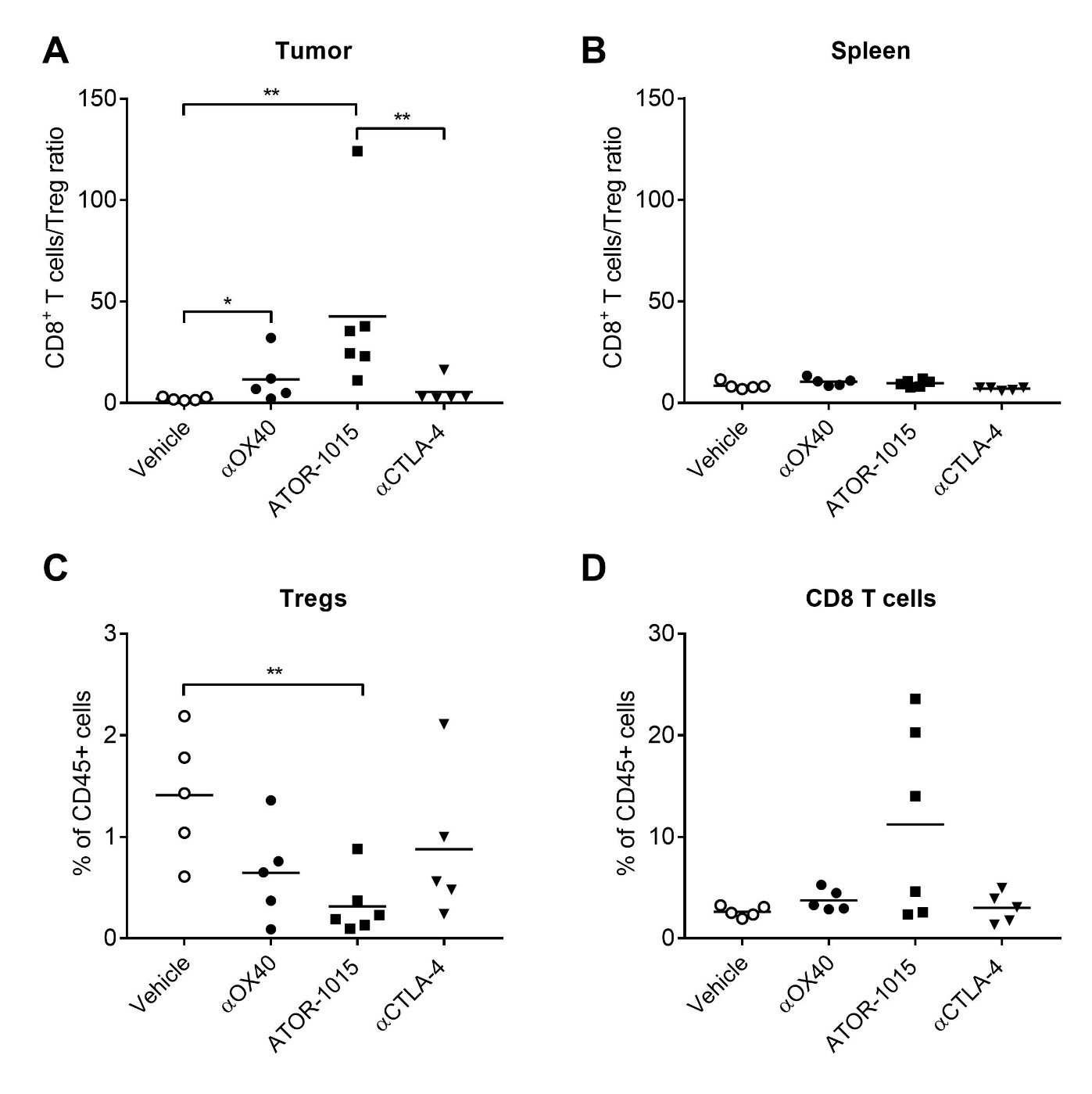


**Figure S3.** **ATOR-1015 depletes Tregs and activates effector T cells in the tumor.** Homozygous hOX40tg mice were inoculated with MB49 cells day 0 and treated ip with ATOR-1015, monotargeting anti-CTLA-4 and anti-OX40 antibodies (248 µg for bsAbs or 200 µg for mAbs) or vehicle on days 14, 17 and 20. Twenty-four hours after the last injection, the tumors and spleens were harvested, and stained for Treg and effector T cell markers. **(A-B)** CD8^+^ T cell/Treg ratio in the tumor and spleen. **(C)** Percentage of Tregs (of CD45^+^ cells) in tumors. **(D)** Percentage CD8^+^ T cells (of CD45^+^ cells) in tumors. All data presented as mean and each dot represents one animal. Statistical differences were analyzed using Mann-Whitney, two-tailed test, (*, p<0.05; **, p<0.01).
