# Supplementary material for: The CTLA-4 x OX40 bispecific antibody ATOR-1015 induces anti-tumor effects through tumor-directed immune activation
Source: J Immunother Cancer. 2019 Apr 11;7:103. doi: 10.1186/s40425-019-0570-8 (PMC6458634; doi:10.1186/s40425-019-0570-8)
Supplement: Supplementary file 6 — Figure S4. ATOR-1015 improves the effect of PD-1 blocking antibodies. (DOCX 206 kb) [file 40425_2019_570_MOESM6_ESM.docx]

Additional file 6: Figure S4

| **Treatment** | **CT26 – tumor growth** | **CT26 - survival** |
| --- | --- | --- |
| Vehicle *vs* ATOR-1015 | p=0.0008 (***) | p=0.0145 (*) |
| Vehicle *vs* ATOR-1015 + PD-1 | p<0.0001 (****) | p<0.0001 (****) |
| PD-1 *vs* ATOR-1015 | p=0.0172 (*) | p=0.0186 (*) |
| PD-1 *vs* ATOR-1015 + PD-1 | p=0.0001 (***) | p<0.0001 (****) |
| **Treatment** | **MC38 – tumor growth** | **MC38 - survival** |
| Vehicle *vs* ATOR-1015 | p=0.001 (***) | p<0.0001 (****) |
| Vehicle *vs* ATOR-1015 + PD-1 | p=0.0362 (****) | p=0.0023 (**) |
| PD-1 *vs* ATOR-1015 | p<0.0001 (****) | p<0.0001 (****) |
| PD-1 *vs* ATOR-1015 + PD-1 | p<0.0001 (****) | p=0.0002 (***) |
| ATOR-1015 *vs* ATOR-1015 + PD-1 | p=0.0028 (**) | p=0.0026 (**) |

**Figure S4. ATOR-1015 improves the effect of PD-1 blocking antibodies.** Female hOX40tg mice, either heterozygotes (CT26, n=18) or homozygotes (MC38, n=26), were inoculated sc with tumor cells on day 0 and then treated ip with 248 µg ATOR-1015, and/or 250 µg anti-PD-1 (RPM1-14) or vehicle on days 7, 10 and 13. Tumor volume and survival were followed over time. Tumor volume is presented as mean ± SEM. Statistical differences were analyzed using Mann-Whitney, two-tailed test for tumor growth, and Kaplan-Meier, Log-Rank for survival and is presented in the table (*, p<0.05; **, p<0.01; ***, p<0.001; ****, p<0.0001). n equals the number of mice.
