# Supplementary material for: The CTLA-4 x OX40 bispecific antibody ATOR-1015 induces anti-tumor effects through tumor-directed immune activation
Source: J Immunother Cancer. 2019 Apr 11;7:103. doi: 10.1186/s40425-019-0570-8 (PMC6458634; doi:10.1186/s40425-019-0570-8)
Supplement: Supplementary file 7 — Figure S5. Anti-tumor effect of ATOR-1015 in hOX40tg mice bearing CT26 colon carcinoma. (DOCX 100 kb) [file 40425_2019_570_MOESM7_ESM.docx]

Additional file 7: Figure S5

***
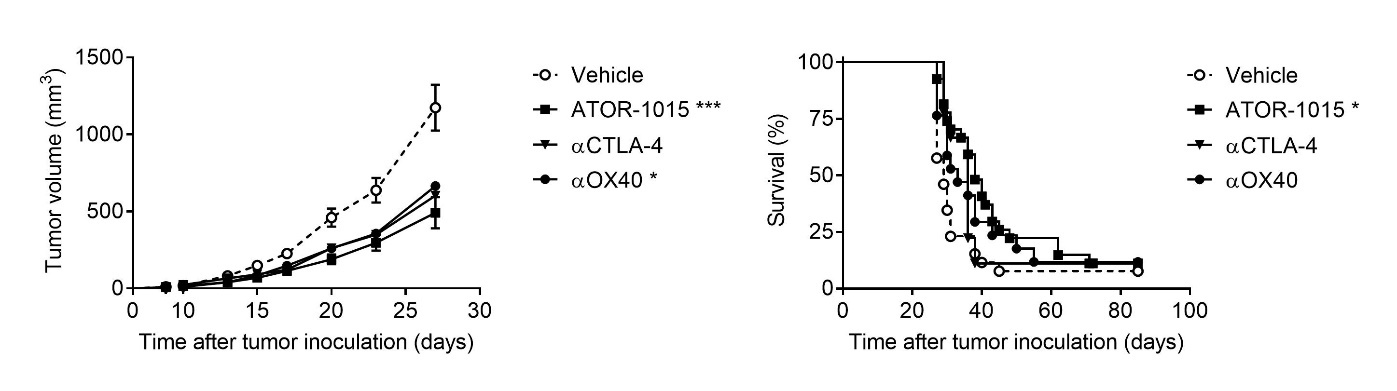
***

**Figure S5. Anti-tumor effect of ATOR-1015 in hOX40tg mice bearing CT26 colon carcinoma.** Female heterozygous hOX40tg mice were inoculated sc with CT26 tumor cells on day 0. Administration ip of ATOR-1015, monotargeting (anti-OX40 and anti-CTLA-4) antibodies (200 µg for mAbs and 248 µg for bsAbs) or vehicle was performed on days 7, 10 and 13 (n=28 mice). Tumor volume as mean +/- SEM. Statistical differences compared to vehicle were analyzed using Mann-Whitney, two-tailed test for tumor growth and Kaplan-Meier, Log-Rank for survival (*, p<0.05; ***, p<0.001).
