# Supplementary material for: The CTLA-4 x OX40 bispecific antibody ATOR-1015 induces anti-tumor effects through tumor-directed immune activation
Source: J Immunother Cancer. 2019 Apr 11;7:103. doi: 10.1186/s40425-019-0570-8 (PMC6458634; doi:10.1186/s40425-019-0570-8)
Supplement: Supplementary file 8 — Figure S6. Anti-tumor effect of ATOR-1015 in hOX40tg mice bearing PANC02 pancreas cancer. (DOCX 103 kb) [file 40425_2019_570_MOESM8_ESM.docx]

Additional file 8: Figure S6

**
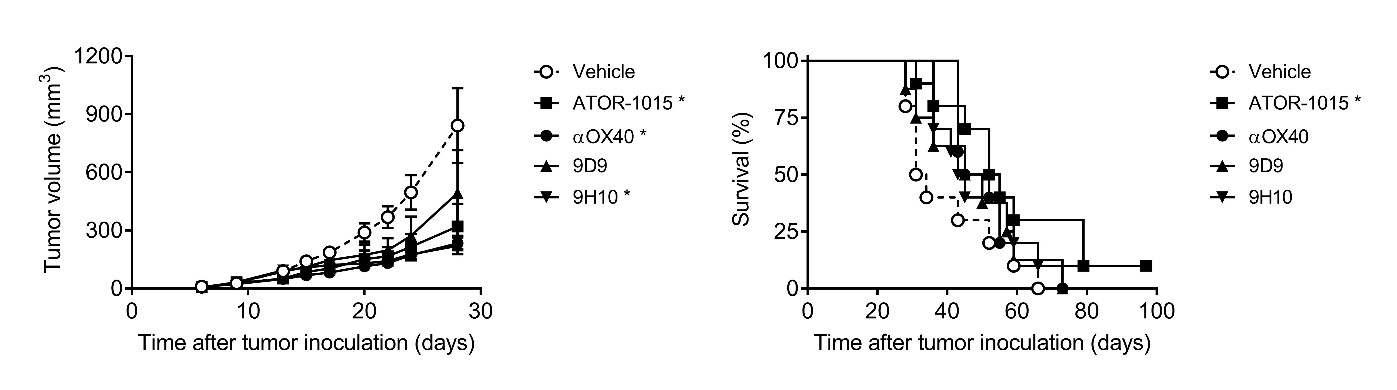
**

**Figure S6.** **Anti-tumor effect of ATOR-1015 in hOX40tg mice bearing PANC02 pancreas cancer.** Female heterozygous hOX40tg mice were inoculated sc with PANC02 tumor cells on day 0. Administration ip of ATOR-1015, commercial anti-CTLA-4 antibodies (9D9 and 9H10), anti-OX40 antibody (200 µg for mAbs and 248 µg for bsAbs) or vehicle was performed on days 6, 9 and 13 (n=10 mice). Tumor volume as mean +/- SEM. Statistical differences compared to vehicle were analyzed using Mann-Whitney, two-tailed test for tumor growth and Kaplan-Meier, Log-Rank for survival (*, p<0.05).
