# Supplementary material for: The CTLA-4 x OX40 bispecific antibody ATOR-1015 induces anti-tumor effects through tumor-directed immune activation
Source: J Immunother Cancer. 2019 Apr 11;7:103. doi: 10.1186/s40425-019-0570-8 (PMC6458634; doi:10.1186/s40425-019-0570-8)
Supplement: Supplementary file 9 — Figure S7. Non-responsive models. (DOCX 117 kb) [file 40425_2019_570_MOESM9_ESM.docx]

Additional file 9: Figure S7


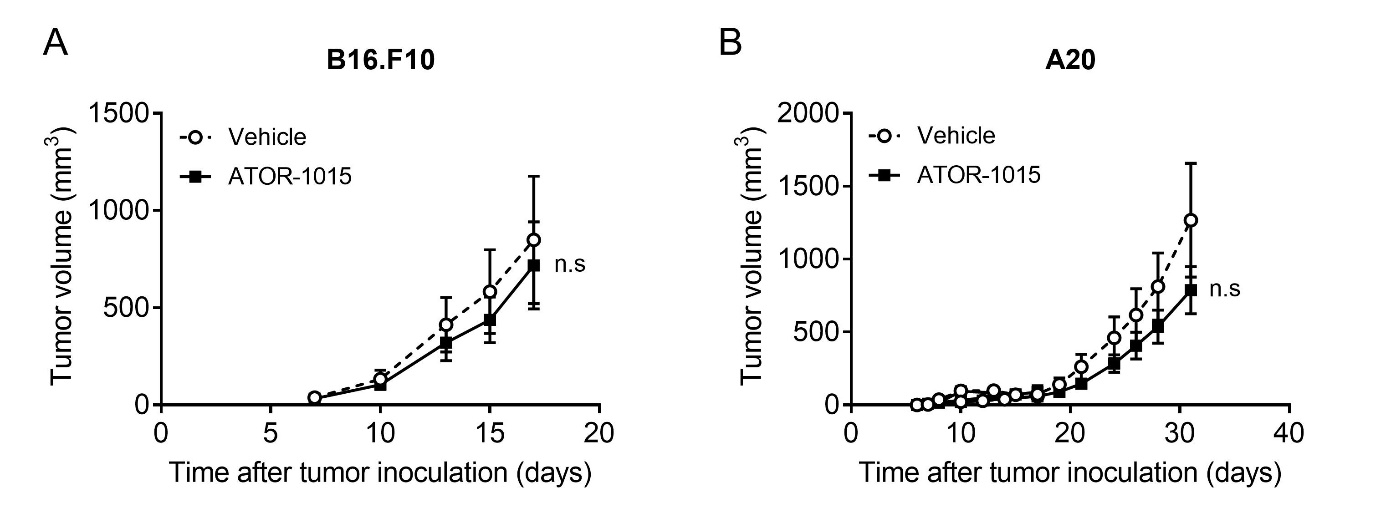


**Figure S7.** **Non-responsive models.** Female hOX40tg mice were inoculated sc with tumor cells on day 0 and treated with vehicle or ATOR-1015 (248 µg) on days 7, 10 and 13. **(A)** Heterozygous mice with B16.F10 melanoma (n=10 mice). **(B)** Homozygous mice with A20 lymphoma (n=7-9 mice). Tumor volume as mean +/- SEM. Statistical differences compared to vehicle were analyzed using Mann-Whitney, two-tailed test (n.s, not significant).
